# Error estimation and compensation in 4R and P3R closed-chain mechanisms due to joint clearance: a comparative study

**DOI:** 10.1038/s41598-025-19941-4

**Published:** 2025-10-15

**Authors:** Ankur Jaiswal, Darren Alton Dsouza, H. P. Jawale, Abhishek Jha, Anil Kumar, Munendra Singh

**Affiliations:** 1 Department of Mechatronics, Manipal Institute of Technology, Manipal Academy of Higher Education (MAHE), Manipal, 576104 Karnataka India; 2 Centre for Product Design and Manufacturing, Division of Mechanical Engineering, Indian Institute of Science, Bangalore, 560012 India; 3 Department of Mechanical Engineering, Visvesvaraya National Institute of Technology, Nagpur, 440010 India; 4 Department of Mechanical Engineering Amrita School of Engineering, Amrita Vishwa Vidyapeetham, Chennai, India; 5 Department of Mechanical Engineering, Bhilai Institute of Technology, Durg, Chhattisgarh India; 6 Department of Electronics and Communication Engineering, National Institute of Technology Jamshedpur, Jamshedpur, Jharkhand India

**Keywords:** Kinematic formulation, R-type and P-type mechanisms, Mathematical approach, CAD model, Joint clearance, Validation, Trajectory generation, Mechanical engineering, Engineering, Mathematics and computing

## Abstract

Four-bar linkages form the fundamental configuration of many planar mechanisms, and joint clearance is one of the primary factors that introduce deviations from their intended output. This paper presents a performance assessment of planar mechanisms with prismatic (P) and revolute (R) joints specifically 4R and P3R configurations under the influence of joint clearance. A detailed methodology for mechanical error analysis and compensation is employed. Both mechanisms are evaluated for identical trajectory generation tasks to ensure a fair comparative analysis. It is found that joint clearance leads to non-uniform positional errors across the mechanism’s working range, contrary to common assumptions of uniform error. Notably, the 4R mechanism exhibits greater robustness and lower sensitivity to joint clearance-induced positional errors compared to the P3R configuration. These findings suggest that revolute joint-based actuation is preferable to prismatic actuation for minimizing positional inaccuracy in robotic manipulators. The proposed error compensation framework also provides a generalized approach for assessing and improving the performance of mechanisms affected by mechanical inaccuracies.

## Introduction

Planar closed-chain mechanisms are one of the most significant fields of robotics and automation study today. To position tools or things where they are needed, robotic manipulators are frequently utilized. Several applications employ closed-chain mechanisms with spatial and planar configurations as manipulators. A common example of such a mechanism is the four-bar linkage, which is employed in many machines and processes to produce the desired path, function, and motion. Applications for four bar mechanisms and its inversion include machine linkages, automobiles, biomedical equipment, and many more. To explore a variety of complex processes, a straightforward structure like a four-link chain is employed. The type of input condition of the mechanism has an impact on the performance metrics of the mechanism, such as positioning accuracy, structural and mechanical defects^1,2^. Such manipulators might be used for quick automations since they are more affordable than traditional open chain robots. The main stages of such applications include simple planar mechanisms and various inversions developing same coupler trajectory. A coupler point may be used as an end effector and the closed chain mechanism can be created as a manipulator by using the perfect drive. It is inevitable that joints in mechanisms will have clearance. It takes a very little, adequate clearance for a mechanism to move smoothly. On the other side, joint clearance has negative consequences that are seen. A common concept for mechanism joint clearance among researchers is an extra link with a length equal to half the joint clearance^3–8^. These mechanisms employ the R-type and P-type actuators to accomplish the required task. The selection of an actuator is determined by the functional requirements of kinematic and dynamic parameters for the mechanisms. As a result, when choosing between linear and rotating actuation, a comparative performance analysis is essential. The essential performance feature for such research is the positional inaccuracy caused by structural and mechanical elements. To discover and improve a mechanism’s accuracy and precision, positional error analysis is essential. This facilitates the reduction of the structural features of the mechanism and the investigation of the input–output relationship. Additionally, this research helps to comprehend and eliminate manufacturing flaws and defects^3,9–13^.

Joint clearances are a result of the fact that minute manufacturing flaws can introduce angular or linear deviations into a mechanism as it operates, leading to minor errors. Think about the P3R mechanism’s revolute joint. One of the most common forms of joints found in planar mechanisms i.e. pin joint or hinged joint, which is used in this junction^5^. Since these errors are unpredictable, it is very difficult and expensive to foresee and rectify them. Considering the slot as the central point, we can imagine an extra link that creates a circle with a slot as the center. This connection serves as the clearance or joint clearance^14^. To assess its effect on dynamic properties, positional deviation, vibrations and noise, accelerations, and surface wear, mechanism joint clearance was represented as an additional link with a length equal to half of the joint clearance. The position and direction of the clearance vector are affected by a few dynamic factors, including the speed of operation, inertia forces, and load on mechanisms. Joint clearance causes a positional change that is greater than the combined effect of all the other factors. Since the positional fluctuation it causes is unanticipated and arbitrary, it is vital to investigate its effects^3,7^.

Erkaya et al.^15^ examined the effects of clearance and link flexibility on stresses. Sharfi and Smith investigated the dimension deviation and play in the joints of the complex mechanisms^16^. In multi-loop processes, revolute and prismatic pairs’ joint clearances and the related uncertainty were modelled and analyzed, according to K. L. Ting et al.^17^. Flores^18^ developed a general paradigm for evaluating the effects of manufacturing and assembly tolerance-related kinematic position errors in open and closed chain planar mechanisms. To determine the influence of joint clearance on pose deviation in trajectory, Ting et al. presented the N-bar rotatability principles and advanced optimization techniques^19^. Numerous methods, including the stochastic approach^20,21^, probabilistic model^22^, loop closure technique^23^, and genetic algorithm^24^, are employed in the literature to address issues with positional inaccuracy, drive performance and transmission angle of serial and closed mechanisms. The probability approach is used in serial, planar, and spatial robotics to evaluate the influence of link tolerance and joint clearance on^17,23–27^. Zhang and Xianmin^28^ examined the uncertainty under the clearance on the joints of planar parallel mechanisms. Chen et al.^29^ presented a comparison of the two mechanisms based on position deviation. Jawale and Thorat^30^ investigated the 4R, 2-serial, and P3R processes, as well as the consequences of clearances and backlash. To assess motion sensitivity, mechanical parameters of coupler curves is carried out, as investigated by Erkaya et al.^31^. Due to the joint clearance, Erkaya et al.^32^ examined the kinematic and dynamic performance of the single-DOF planar mechanisms. Tsai and Lai evaluated the multi-link system’s kinematic position and accuracy using the wrench screw method. Joint clearance findings are contrasted with those of the ideal mechanism^33^. Jawale and Thorat^34^ examined the position accuracy of serial and closed chain manipulators, compared the angular errors with joint clearances. Li et al. proposed the geometric technique, an optimization strategy is employed to estimate the orientation errors and verified by Monte Carlo simulations^35^. Tsai and Lai investigated the effects of joint clearances on transmission quality and mechanism faults^36^. Wu and Rao adopted the interval approach to represent tolerances and clearances and to analyse fuzzy errors in mechanisms. The interval number and conventional method are contrasted^37^. By considering the unpredictability of connection lengths and using saddle point approximation to determine the error inside a sphere with a radius equal to the expected error, Zhang and Han proposed a reliable approach^38^. Jaiswal and Jawale et al. investigated mechanical error in four bar revolute joint mechanisms under the impact of line tolerance^39,40^.

This study investigates two mechanisms: the 4R mechanism, consisting solely of revolute joints, and the P3R mechanism, which integrates prismatic and revolute joints. These serve as representative examples of R-type and P-type actuated systems. A comparative analysis is carried out to understand how joint clearance influences each type. The work examines the maximum positional and orientation errors in both mechanisms under varying clearance conditions. For input positions with common trajectory conditions, the study evaluates and contrasts the two actuation modes to identify the more suitable option. The paper is organized into the following sections: methodology, mathematical modeling of closed-chain mechanisms with joint clearance, error compensation through inverse kinematics, results and discussion, and finally, conclusions.

## Methodology

**Fig. 1:**
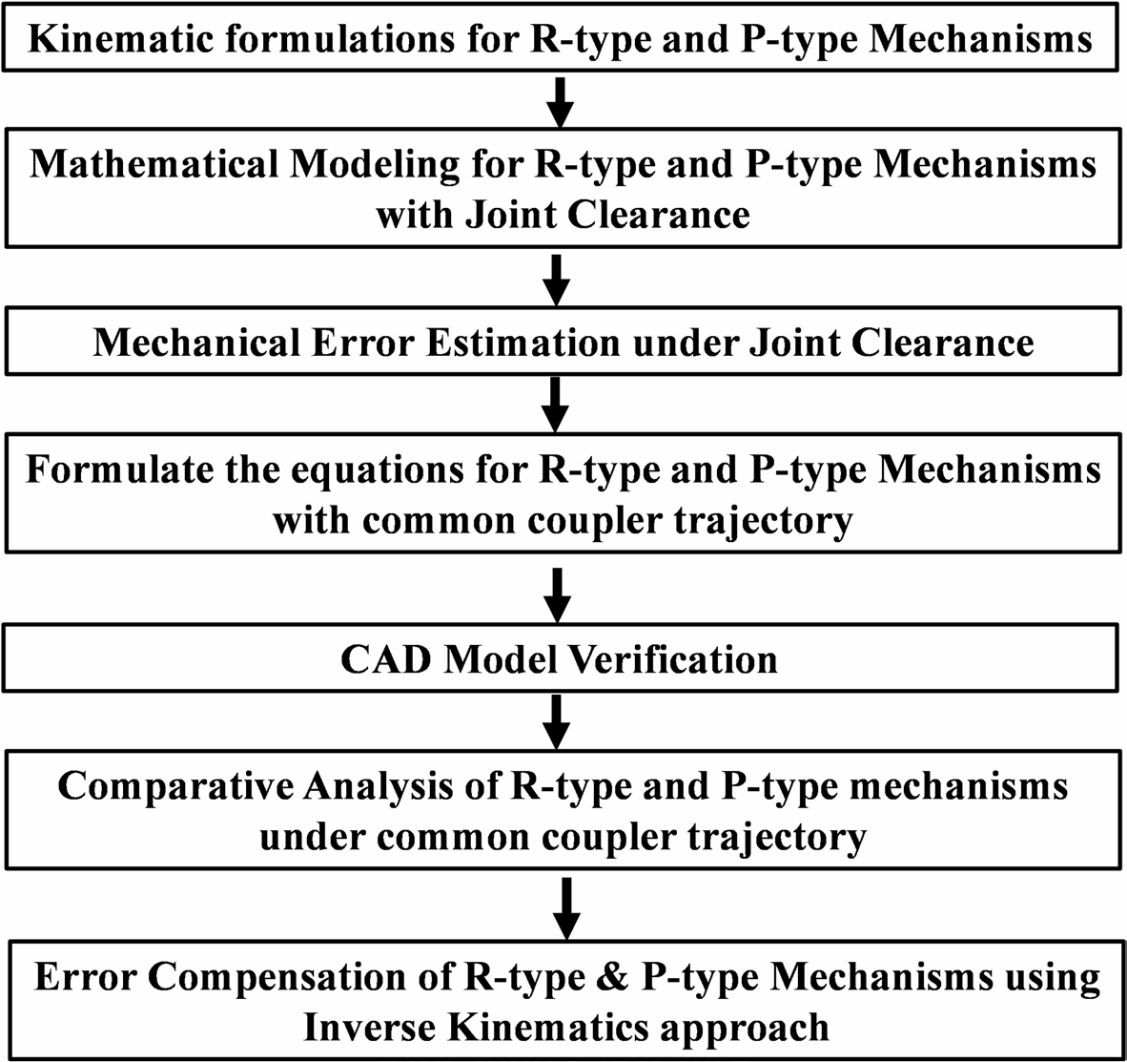
Flow chart.

The flow of the proposed work R-type and P-type mechanisms is examined for positional error and compensation with the effect of joint clearance under identical operating conditions, Fig. 1. The formulation of kinematic equations of R-type and P-type mechanisms is conducted mathematically, estimating common coupler position of given input positions (linear and rotatory) to generate the trajectory for both mechanisms. To estimate and compensate for positional and orientation errors in R-type (4R) and P-type (P3R) closed-chain mechanisms under the influence of joint clearance. The methodology, as illustrated in Fig. 1, consists of several key stages outlined below:

The proposed mathematical model has been verified using the CAD model approach. Furthermore, the effect of joint clearance is incorporated into the kinematic model to examine the maximum positional error obtained and verification. The position and orientation error of the mechanism is examined and evaluated the error compensation using inverse kinematic approach.

### Mechanism selection and kinematic modelling

Two types of closed-chain planar mechanisms namely the 4R (revolute) mechanism and the P3R (prismatic-revolute) mechanism were selected for analysis due to their common usage in robotic manipulators and parallel mechanisms.

1. Kinematic equations for both mechanisms were derived based on loop-closure equations using standard vector and transformation matrix techniques.
2. The forward kinematics were used to compute the desired coupler position (trajectory) corresponding to 11 pre-defined input configurations for each mechanism (rotational inputs for 4R and linear inputs for P3R).

### Introduction of joint clearance

To simulate real-world uncertainties, joint clearance was introduced into the kinematic model:

1. Each revolute or prismatic joint was assumed to have a maximum radial clearance, represented as an offset in joint center, modelled as a radial link of up to 1 mm.
2. These perturbations were incorporated into the mathematical kinematic equations to estimate the deviated (actual) coupler positions under maximum clearance conditions.

### Error estimation

The positional and orientation errors between the desired and deviated (clearance-affected) trajectories were computed as follows:

1. Positional error was quantified as the Euclidean distance between the normal and deviated coupler positions.
2. Orientation error was calculated as the absolute angular difference between the desired and deviated orientation angles of the coupler link.

This analysis was performed for all 11 input positions for both mechanisms.

### CAD model verification

To validate the mathematical models:

1. A detailed CAD simulation was performed. Both desired and clearance-affected trajectories were replicated in the CAD environment.
2. The resulting coupler positions and orientation angles from the CAD model were compared to the values obtained from the mathematical model to ensure consistency and accuracy.

### Error compensation using inverse kinematics

An inverse kinematics-based compensation strategy was implemented to correct for the observed positional errors due to joint clearance:

1. The required adjustment in the actuator input (rotational for 4R, linear for P3R) was calculated to bring the mechanism back to its original desired pose despite the presence of joint clearance.
2. The compensation values were computed for each input position under various clearance levels.
3. Compensation trends were compared between both mechanisms to evaluate their robustness and sensitivity to joint clearance.

### Comparative analysis

A comparative study was conducted between the 4R and P3R mechanisms by evaluating:

1. Maximum positional and orientation errors across the input range.
2. Required input on actuators or drive to compensations for different clearance levels.
3. Overall robustness of the mechanism to joint clearances in terms of both pose deviation and compensation effort.

## Kinematic formulation for R-type and P-type mechanisms

### R-type mechanism

**Fig. 2:**
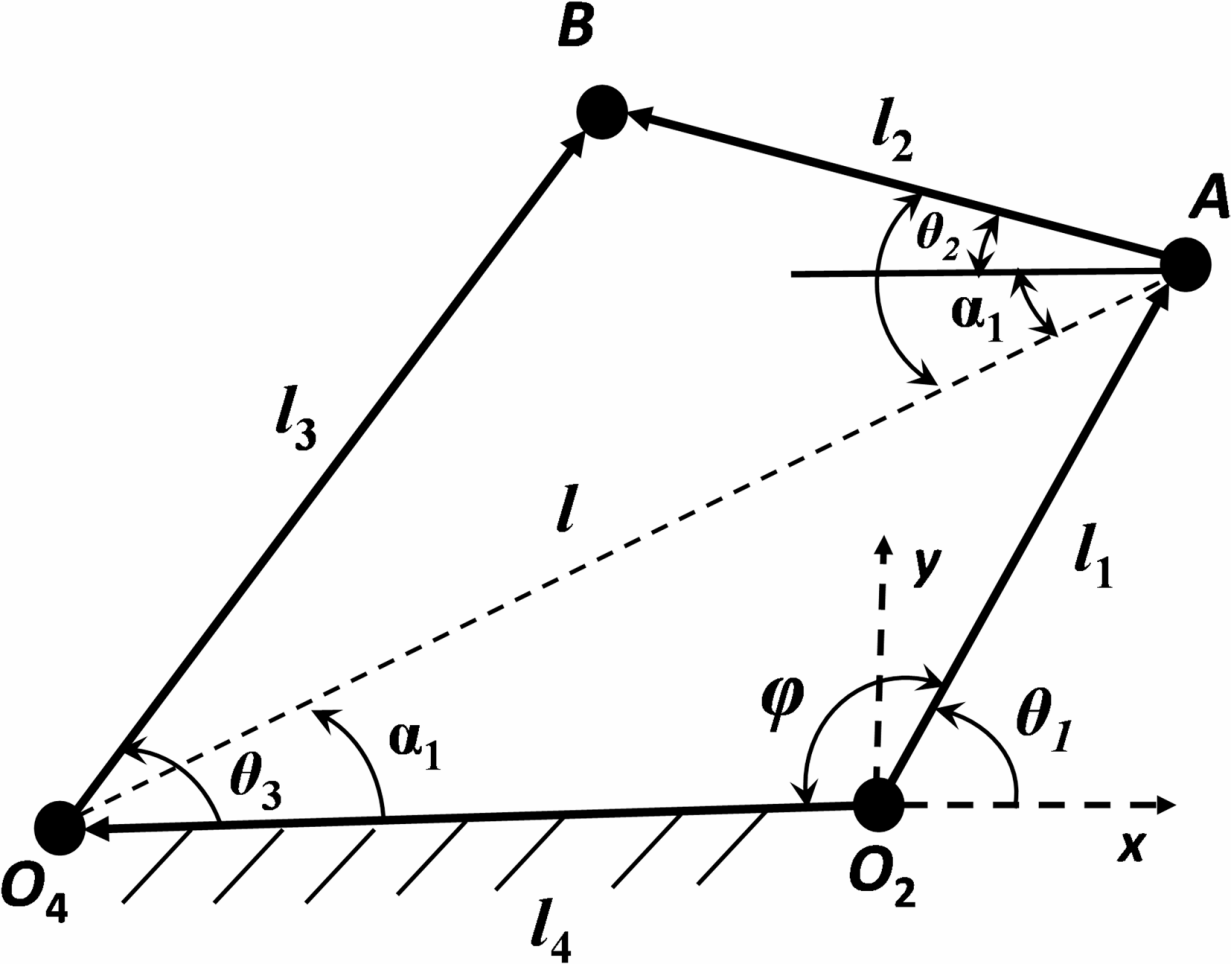
Kinematic sketch of R-type (4R) mechanism.

In Fig. 2, represents a 4R (4-Revolute) mechanism in a task space system. Taking design parameters *l*_1_, *l*_2_, *l*_3_, *l*_4_, *θ*_1_, as inputs and *θ*_3_ as the output angle of displacement equation is written as:

1 $$\varphi = 180 - \theta_{1}$$

2 $$cos\varphi = \frac{{l_{4}^{2} + l_{1}^{2} - l^{2} }}{{2l_{4} l_{1} }}$$

3 $$l = \sqrt {l_{4}^{2} + l_{1}^{2} - 2l_{4} l_{1} cos\varphi }$$

4 $$\alpha_{1} = cos^{ - 1} \left( {\frac{{l_{4}^{2} + l^{2} - l_{1}^{2} }}{{2l_{4} l}}} \right)$$

5 $$\beta_{1} = cos^{ - 1} \left( {\frac{{l_{2}^{2} + l^{2} - l_{3}^{2} }}{{2l_{2} l}}} \right)$$

6 $$\theta_{2} = \beta_{1} - \alpha_{1}$$

7 $$B_{x} = l_{1} cos\theta_{1} - l_{2} cos\theta_{2} = - 100 + 150cos\theta_{2}$$

8 $$B_{y} = l_{1} sin\theta_{1} + l_{2} sin\theta_{2} = 150sin\theta_{3}$$

### P-type mechanism

**Fig. 3:**
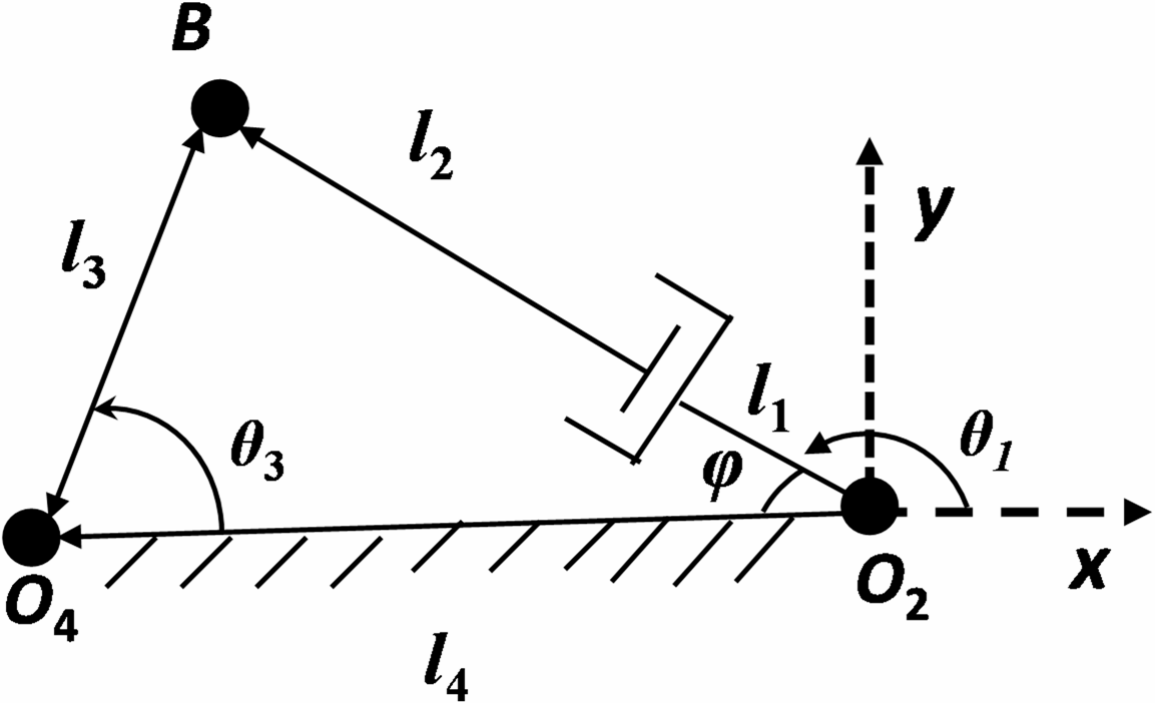
Kinematic sketch of P-type (P3R) mechanism.

In Fig. 3, the planar P3R (1-prismatic and 3-Revolute) mechanism with a linear actuation is demonstrated. The kinematic equation is to determine the coupler position with the variation input displacement through the linear drive motor or actuator.

Let the instantaneous actuator length be *v* given as

9 $$v = \left( {l_{1} + l_{2} } \right) + \delta$$

where ${\updelta }$ = displacement of the actuator.

Using cosine theorem,

10 $$\varphi = cos^{ - 1} \left( {\frac{{v^{2} + l_{4}^{2} - l_{3}^{2} }}{{2l_{4} v}}} \right)$$

11 $$\theta_{3} = cos^{ - 1} \left( {\frac{{l_{3}^{2} + l_{4}^{2} - v^{2} }}{{2l_{3} l_{4} }}} \right)$$

12 $$\theta_{1} = 180 - \varphi$$

13 $$B_{x} = - vcos\varphi = - 100 + 150cos\theta_{3}$$

14 $$B_{y} = vsin\varphi = 150sin\theta_{3}$$

## Mathematical modelling under joint clearance

### Joint clearance

**Fig. 4:**
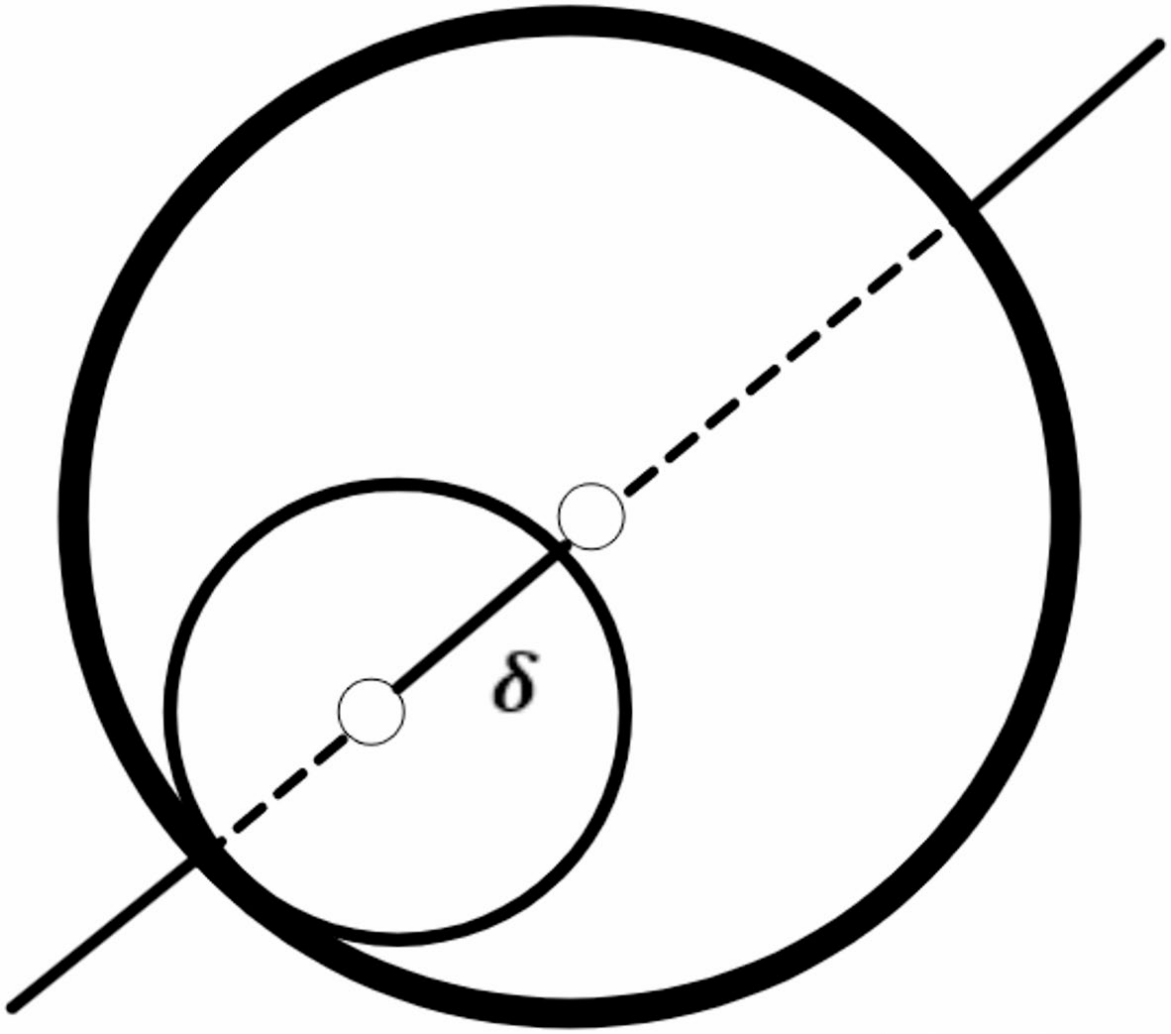
Radial clearance link at joint.

Geometrically, joint clearance (δ) is the difference of radius of pin and the hole as in Fig. 4. A pin joint (or revolute joint) ideally allows pure rotation between two links with no relative translation. Due to manufacturing tolerances or wear, a clearance (gap) exists between the pin and the hole in Fig. 4. In this situation, clearance is defined as a small link (radius of the pin) between nominal links of the mechanism. Each joint with clearance will provide the extra single degree of freedom (DOF) to the mechanism.

### Clearance formulation for R-type mechanism

**Fig. 5:**
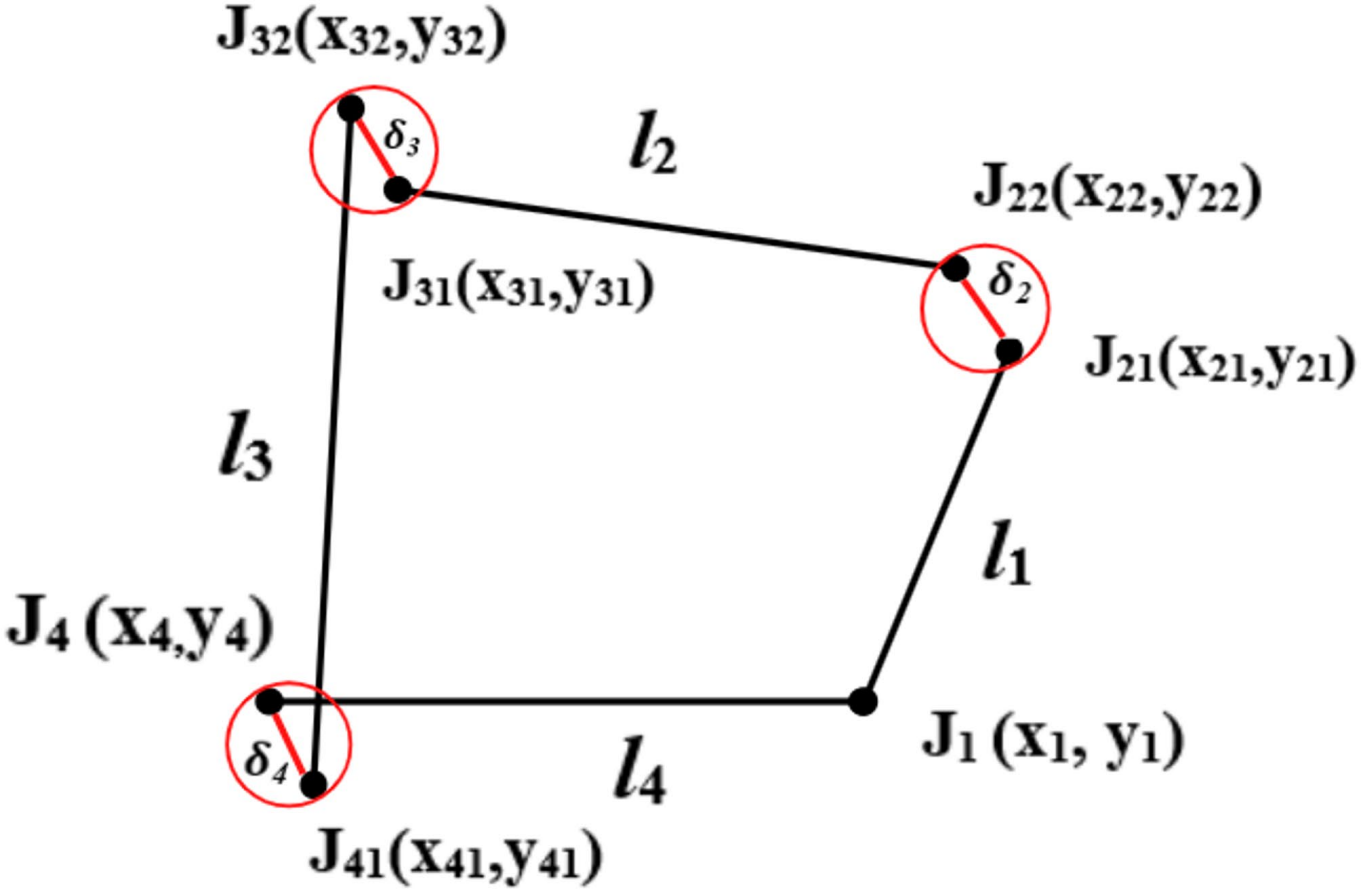
R-type mechanism with joint clearance.

As shown in Fig. 5 coupler point is shifted to J_32_ from J_31_ under effect of joint clearance. Each joint is altered due to clearance link. Position Let *λ*_1_, *λ*_2_, *λ*_3_, and *λ*_4_ are angle made by clearance link about J_1_, J_21_, J_31_ and J_4_ with fixed link axis and *δ*_1_, *δ*_2_, *δ*_3_, and *δ*_4_ are the clearance link lengths, the deviated position of joint J_4_ is J_41_(*x*_41_, *y*_41_) such that,

15 $$x_{21} = l_{1} cos\theta_{1}$$

16 $$y_{21} = l_{1} sin\theta_{1}$$

17 $$x_{22} = x_{21} + \delta_{2} cos\lambda_{2}$$

18 $$y_{22} = y_{21} + \delta_{2} sin\lambda_{2}$$

19 $$x_{31} = x_{22} - l_{2} cos\theta_{2}$$

20 $$y_{31} = y_{22} + l_{2} sin\theta_{2}$$

21 $$x_{32} = x_{31} + \delta_{3} cos\lambda_{3}$$

22 $$y_{32} = y_{31} + \delta_{3} sin\lambda_{3}$$

23 $$x_{41} = x_{4} + \delta_{4} cos\lambda_{4}$$

24 $$y_{41} = y_{4} + \delta_{4} sin\lambda_{4}$$

25 $$Positional Error = \sqrt {\left( {B_{x} - B_{xnew} } \right)^{2} + \left( {B_{y} - B_{ynew} } \right)^{2} }$$

26 $$Orientation Error = \theta_{{3\left( {deviated} \right)}} - \theta_{{3 \left( {desired} \right)}}$$

27 $$\theta_{{3\left( {new} \right)}} = sin^{ - 1} \left( {\frac{{y_{32} }}{{l_{3} }}} \right) or cos^{ - 1} \left( {\frac{{x_{32} }}{{l_{3} }}} \right)$$

The Eqs. (25), (26) and (27) are used to estimate the pose error in the 4R mechanism by the influence of clearance in joint.

### Clearance formulation for P-type mechanism

**Fig. 6:**
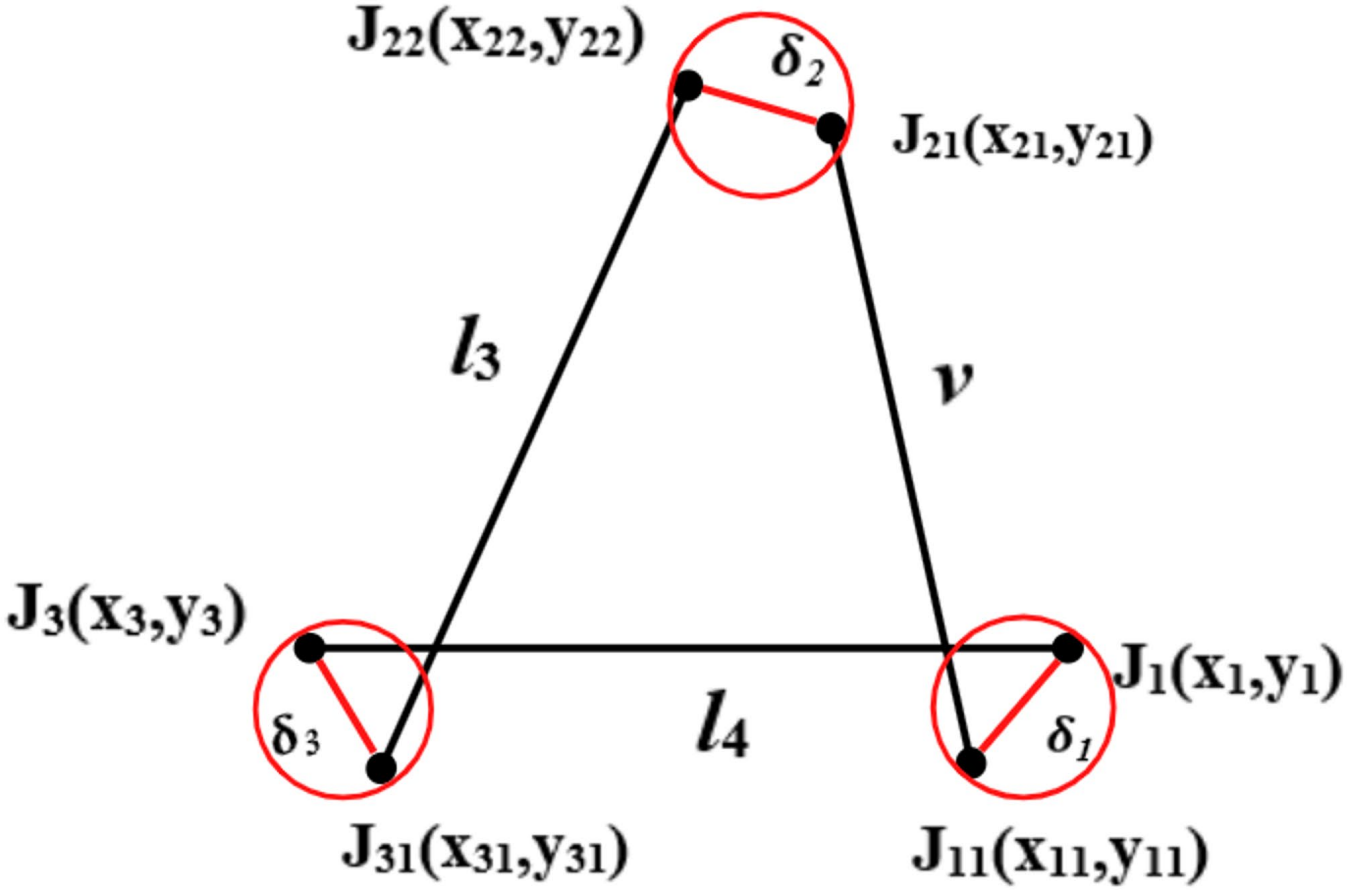
P-type mechanism with joint clearance.

The coupler position is deviated to J_22_ from J_21_ due to joint clearance as shown in Fig. 6. Each joint is affected by the clearance. Position Let λ_1_, λ_2_ and λ_3_ are angle made by clearance link about J_1_, J_21_ and J_31_ with fixed link axis and δ_1_, δ_2_, and δ_3_ are the clearance at joint, the deviated position of joint J_3_ is J_31_(x_31_, y_31_) such that.

28 $$x_{11} = x_{1} + \delta_{1} cos\lambda_{1}$$

29 $$y_{11} = y_{1} + \delta_{1} sin\lambda_{1}$$

30 $$x_{21} = x_{11} + vcos\theta_{1}$$

31 $$y_{21} = y_{11} + vsin\theta_{1}$$

32 $$x_{22} = x_{21} + \delta_{2} cos\lambda_{2}$$

33 $$y_{22} = y_{21} + \delta_{2} sin\lambda_{2}$$

34 $$x_{31} = x_{3} + \delta_{3} cos\lambda_{3}$$

35 $$y_{31} = y_{3} + \delta_{3} sin\lambda_{3}$$

## Common coupler trajectory between R-type and P-type mechanisms

The position error in R-type and P-type mechanisms is caused by clearances on joint deviations and actuators input (rotatory and linear)^38^. Both mechanisms (R-type and P-type) are generated to the same coupler trajectory and the necessary inputs of actuators are estimated.

**Fig. 7:**
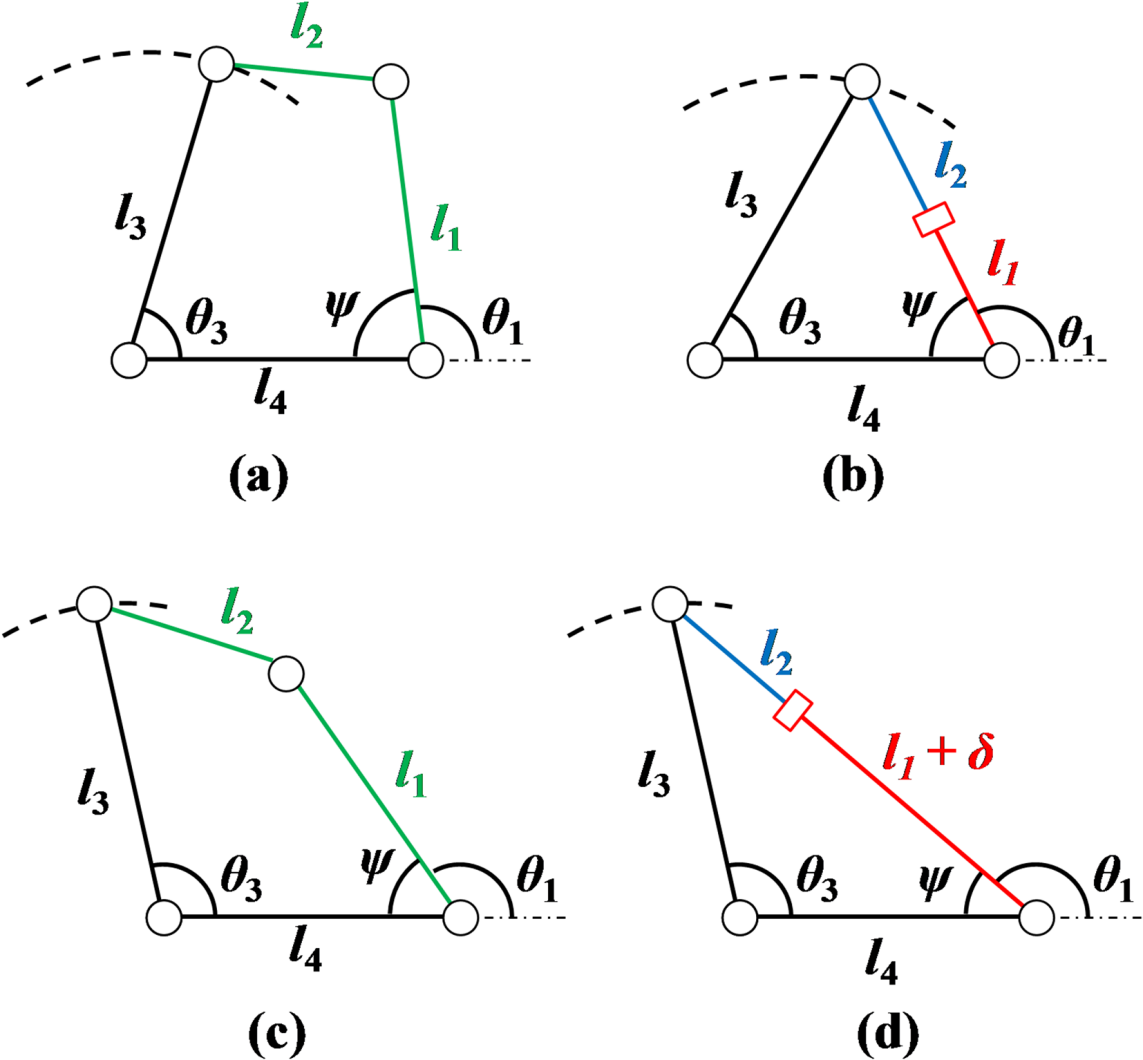
R-type (a, c) and P-type (b, d) mechanisms under common coupler trajectory^38^.

The Fig. 7 (a-b) illustrates the kinematic representation of R-type and P-type mechanisms. Link 1 shows the rotary and linear inputs of R-type and P-type mechanisms. The common coupler trajectory generates for both mechanisms by Link 3 i.e. both mechanisms are subjected to common coupler trajectory is shown in Fig. 7 (c-d). The initial and final position of P-type mechanism with zero and maximum input displacement are shown in Fig. 7(b-d). The equivalent R-type mechanism for output identical or common coupler trajectory to the P-type mechanism.

**Fig. 8:**
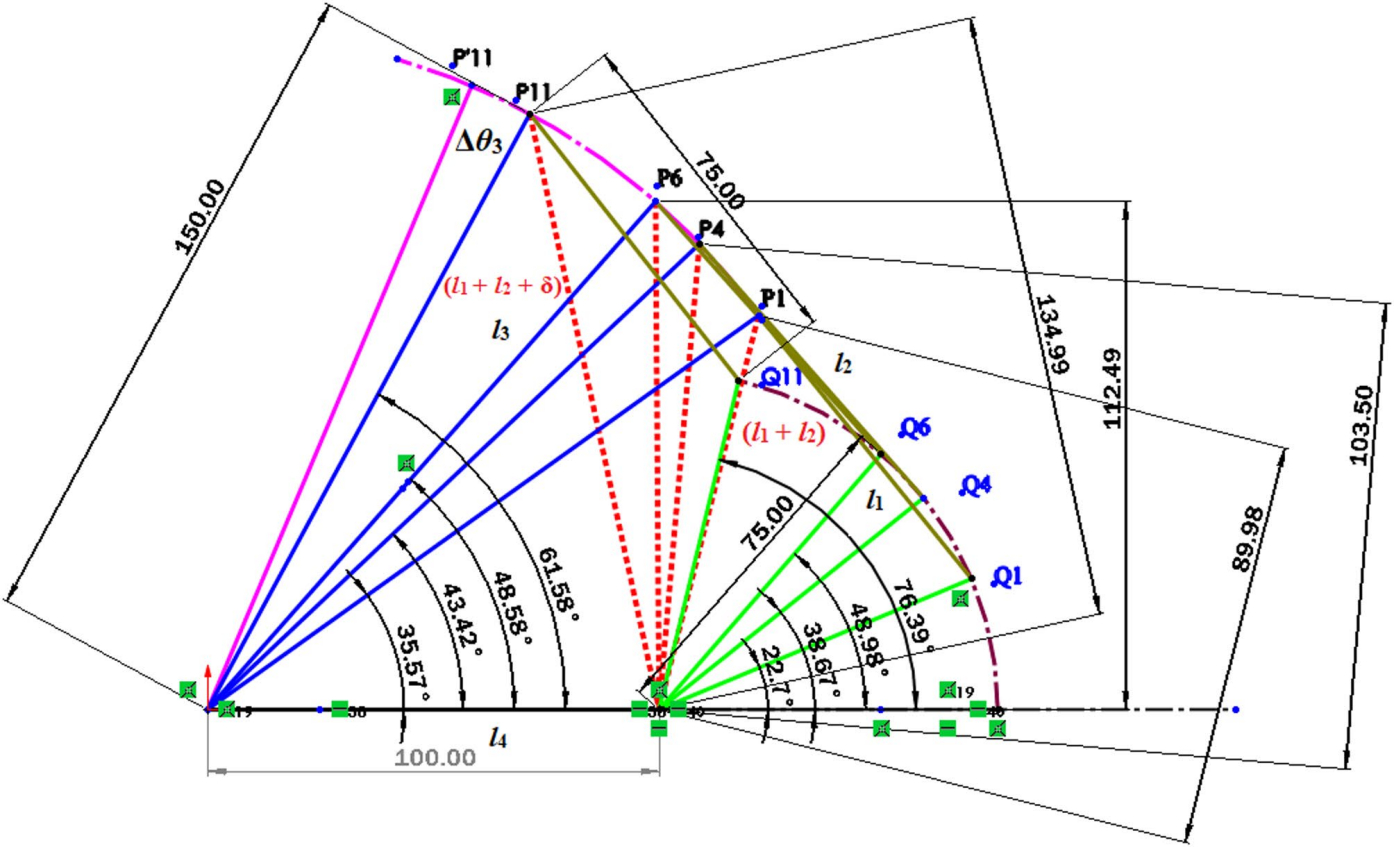
CAD model for common coupler trajectory generation in P3R and 4R mechanisms.

Jaiswal and Jawale^38^ define the mathematical model for common coupler trajectory in both mechanisms (Table 1). The equivalent operating conditions obtained for positions P1 and P11 by mathematically is validated using CAD model approach as seen in Fig. 8.

**Table 1:** Common coupler trajectory for P3R and 4R mechanisms^38^.

Linear displacement in P-type mechanism (*ν*)	Common coupler position	Angular displacement in R-type mechanism (*θ*_1_ deg.)
90	P1	22.7
94.5	P2	28.16
99	P3	33.46
103.5	P4	38.67
108	P5	43.83
112.5	P6	48.98
117	P7	54.17
121.5	P8	59.43
126	P9	64.83
130.5	P10	70.45
135	P11	76.39

The equivalent range of inputs for both P-type and R-type mechanisms is established and presented in Table 1. Moreover, the mathematical approach used to obtain the identical operating conditions [52] for positions P1 and P11 is cross validated using a CAD modelling approach, as depicted in Fig. 8. This investigation provides crucial insights into the relationship between linear and angular displacements in these mechanisms, with potential implications in the field of selection of actuator in the manipulator design.

## Mathematical formulation of inverse kinematics for error compensation

### R-type mechanism

**Fig. 9:**
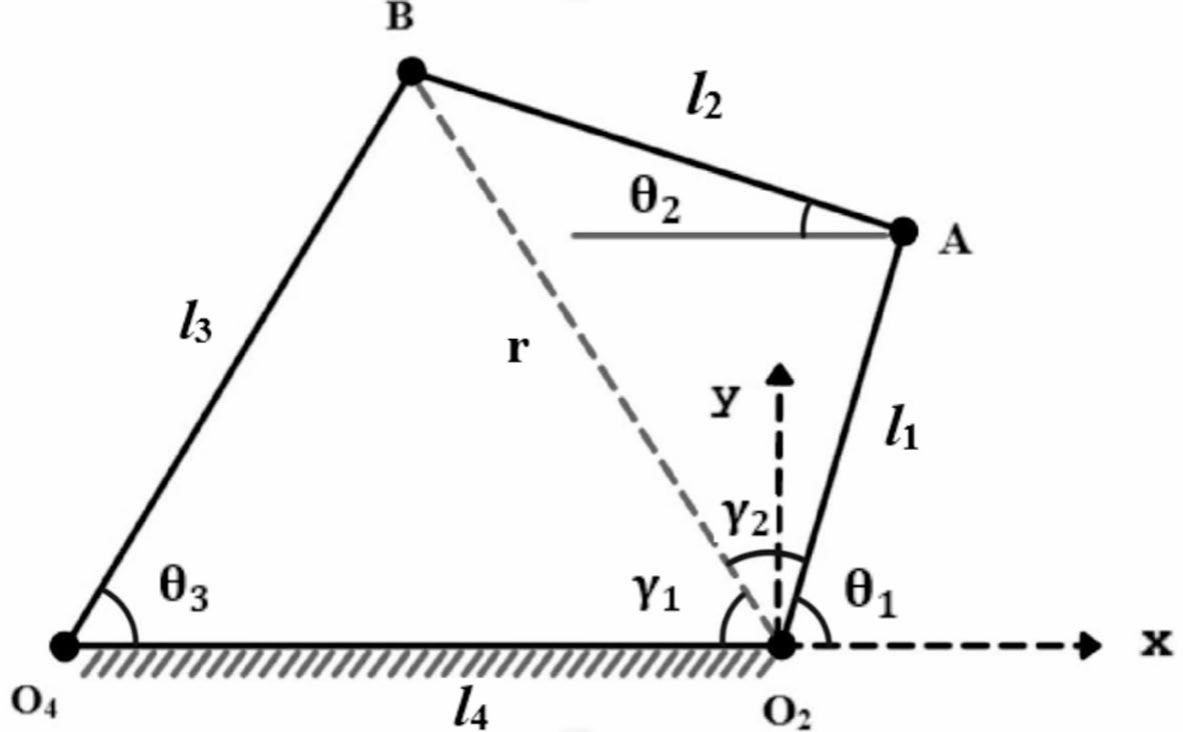
R-type mechanism using inverse kinematics approach.

The positional error obtained due to the difference in desired position and the position obtained due to joint clearance generated by the mechanism is compensated by inverse kinematics approach (Fig. 9). The equations are as listed below:

36 $$\theta_{3} = sin^{ - 1} \left( {\frac{{y_{32} }}{{l_{3} }}} \right)$$

37 $$\gamma_{1} = cos^{ - 1} \left( {\frac{{r^{2} + l_{4}^{2} - l_{3}^{2} }}{{2rl_{4} }}} \right)$$

38 $$\gamma_{2} = cos^{ - 1} \left( {\frac{{l_{1}^{2} + r^{2} - l_{2}^{2} }}{{2rl_{1} }}} \right)$$

39 $$\varphi = \gamma_{1} + \gamma_{2}$$

40 $$\theta_{1} = 180 - \varphi$$

### P-type mechanism

Inverse kinematics is performed on the P3R mechanism to compensate the error obtained due to joint clearances (Fig. 3). The equations derived are as follows

41 $$x = - vcos\varphi$$

42 $$\varphi = cos^{ - 1} \left( {\frac{ - x}{v}} \right)$$

43 $$\theta_{1} = 180 - \varphi$$

## Dimensional parameters

The design parameters for R-type and P-type mechanisms with common trajectory conditions are given as^38^. The common trajectory generation is considered for giving the required comparison parameters for two distinct manipulators, without which performance evaluation is not possible. Initially, R type manipulators with link lengths and the trajectory generated by this configuration are obtained, followed by inverse analysis for a P type manipulator and actuator displacement is estimated. Additionally, considering all links as rigid, the mechanism can be analyzed without accounting for elastic deformations.

R-type mechanism

44 $$l_{{4}} = { 1}00;l_{{3}} = { 1}.{5 } \times l_{{4}} = { 15}0; l_{{1}} = l_{{2}} = { 75}$$

P-type mechanism

45 $$l_{{4}} = { 1}00;l_{{3}} = { 1}.{5 } \times l_{{4}} =_{{}} {15}0;l_{{1}} = l_{{{2} }} = \delta = { 45};$$

## Results and discussion

The positional and orientation error estimation of P-type and R-type mechanisms is carried out for an uncertain situation due to clearance. The appropriate range of clearances, which could be obtained from the assembly of the links with joints, are an analysis. The estimated deviations are interpreted as a pose error; however, it is possible to determine the normalized clearances effect in general.

### Error estimation in R-type mechanism

**Fig. 10:**
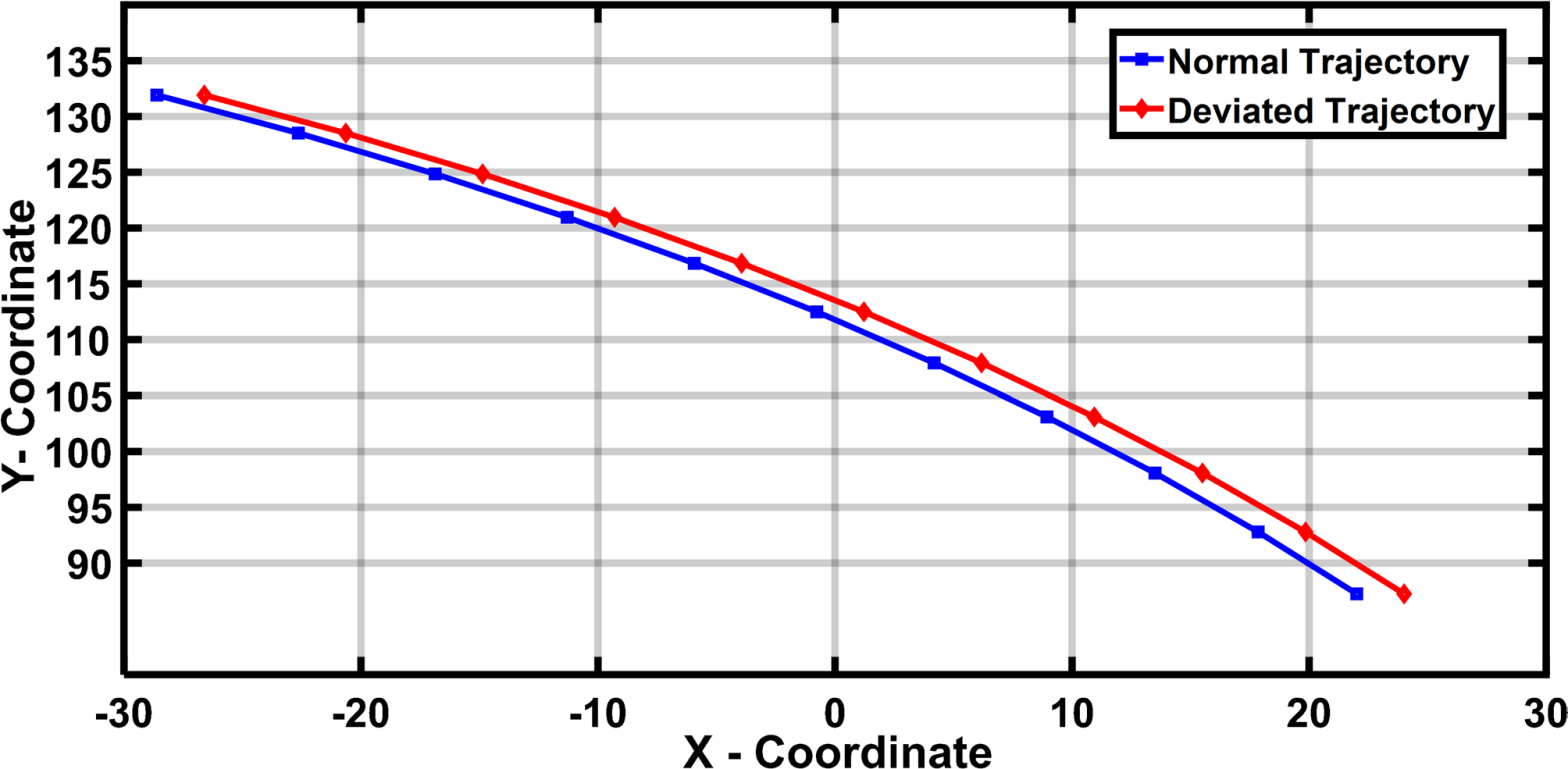
Coupler position in 4R mechanism.

Figure 10 presents the normal and deviated trajectory of the coupler in R-type mechanism with joint clearance in the task space system. The maximum deviated coupler position for 11 rotary actuator inputs is evaluated with the maximum clearance level [52]. There is a deviation in the trajectory generated by the 4R mechanism in Table 2.

**Table 2:** Coordinates of the desired and deviated trajectory for 4R mechanism.

4R mechanism						
S No	Position	Input link position (Degree)	Desired (Normal) coupler position		Deviated coupler position	
			X Co-ordinate	Y Co-ordinate	X Co-ordinate	Y Co-ordinate
1	P1	22.7	22.015	87.248	24.015	87.248
2	P2	28.16	17.851	92.796	19.851	92.796
3	P3	33.46	13.5	98.071	15.5	98.071
4	P4	38.67	8.944	103.108	10.944	103.108
5	P5	43.83	4.186	107.913	6.186	107.913
6	P6	48.98	-0.773	112.49	1.227	112.49
7	P7	54.17	-5.942	116.846	-3.942	116.846
8	P8	59.43	-11.303	120.967	-9.303	120.967
9	P9	64.83	-16.871	124.858	-14.871	124.858
10	P10	70.45	-22.647	128.517	-20.647	128.517
11	P11	76.39	-28.615	131.925	-26.615	131.925

**Fig. 11:**
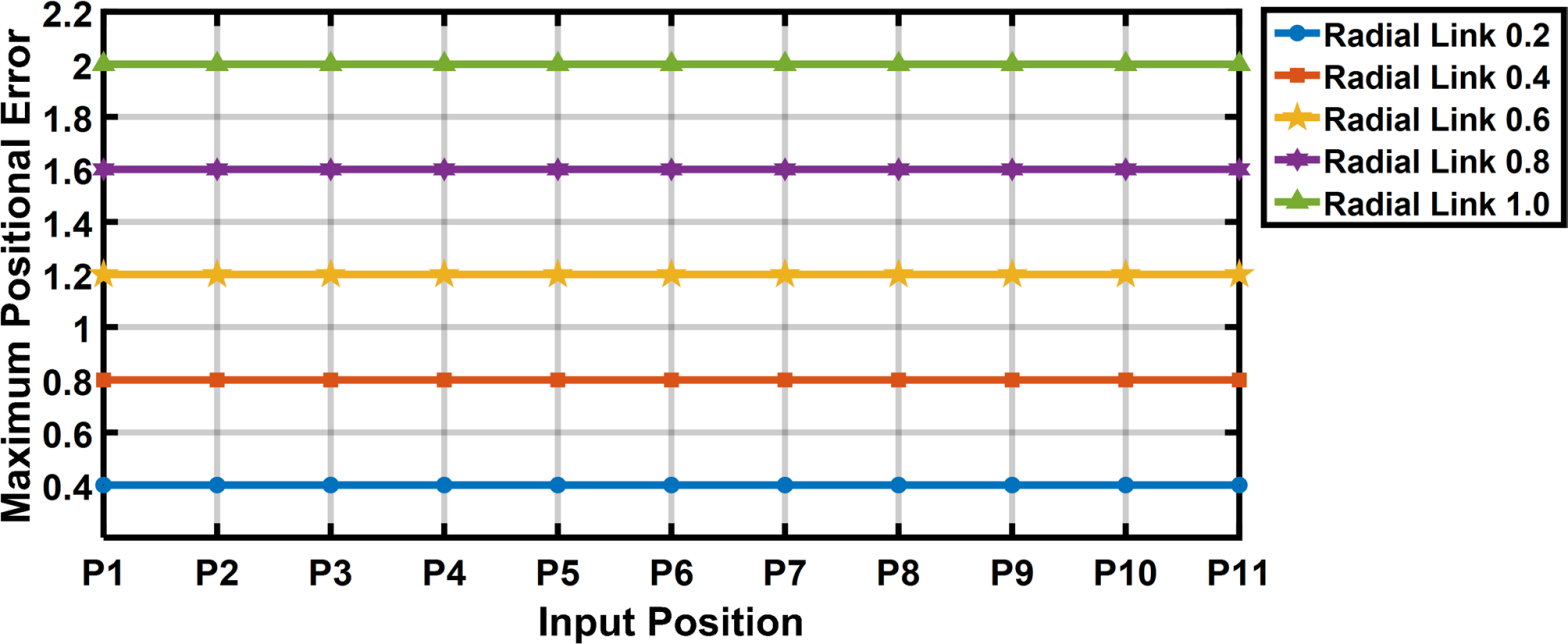
Input link positions vs maximum positional error in R-type mechanism.

**Fig. 12:**
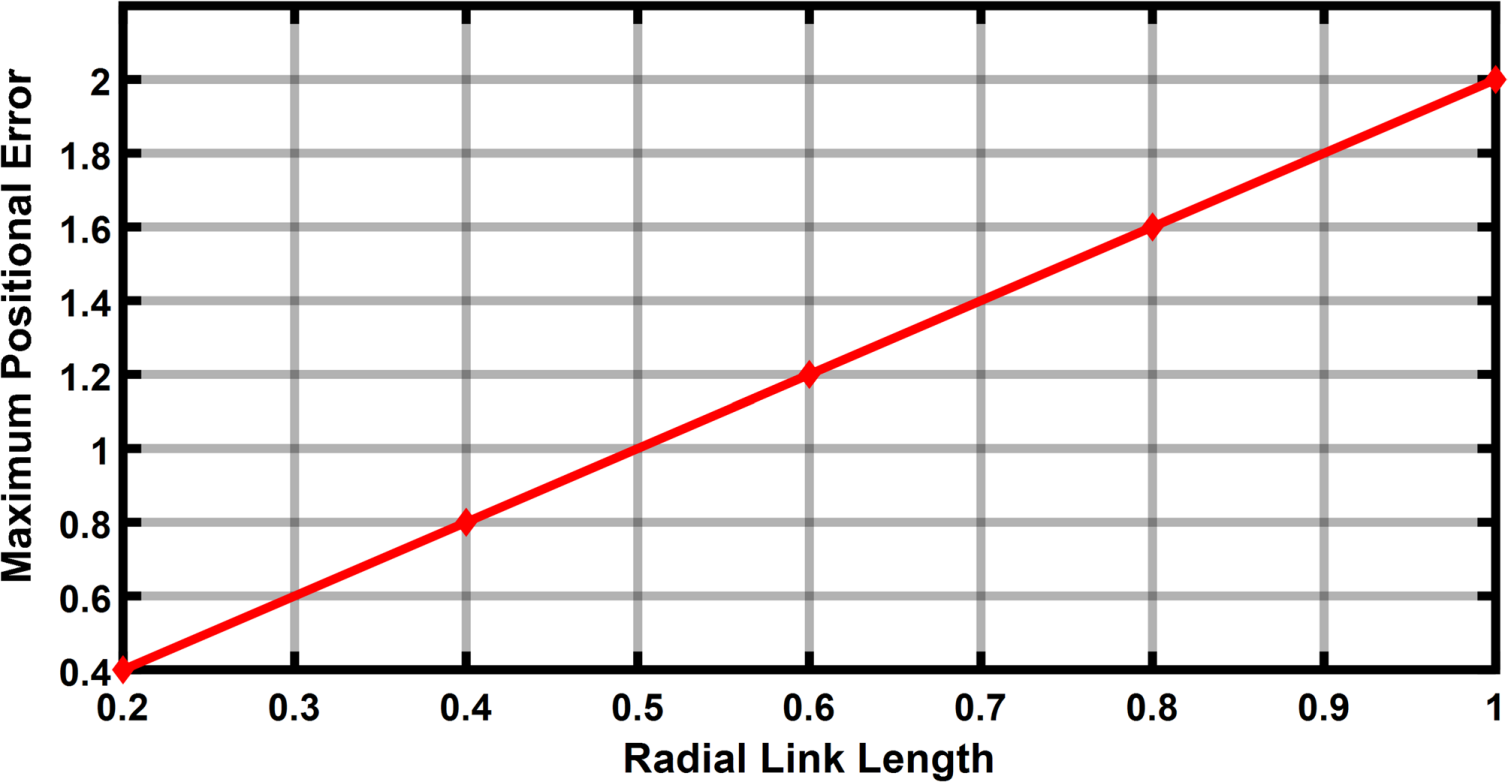
Radial link length vs maximum positional error in R-type mechanism.

The Fig. 11 shows the 11 input link positions versus its maximum positional error with radial link length (joint eccentricity) during joint clearance. As observed through the maximum positional error for a given radial link (joint clearance) for a desired position is twice the radial link length. It is also observed that the Fig. 11 demonstrates radial link (joint clearance) versus maximum positional error relations is a constant. Figure 12 depicted the increase in estimated error with the increase in clearance level. The maximum error obtained is of 2 mm i.e. when the radial link is of 1 mm clearance level.

**Fig. 13:**
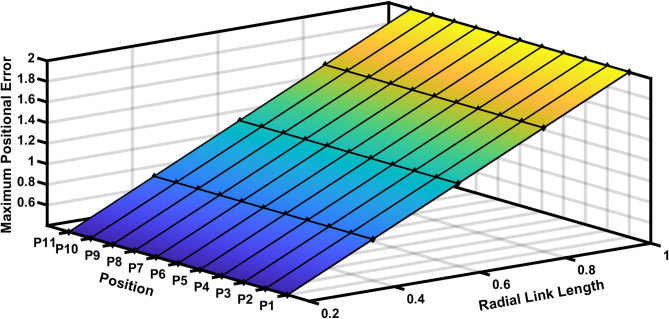
Radial link vs coupler position vs Maximum positional Error in R-type mechanism.

**Fig. 14:**
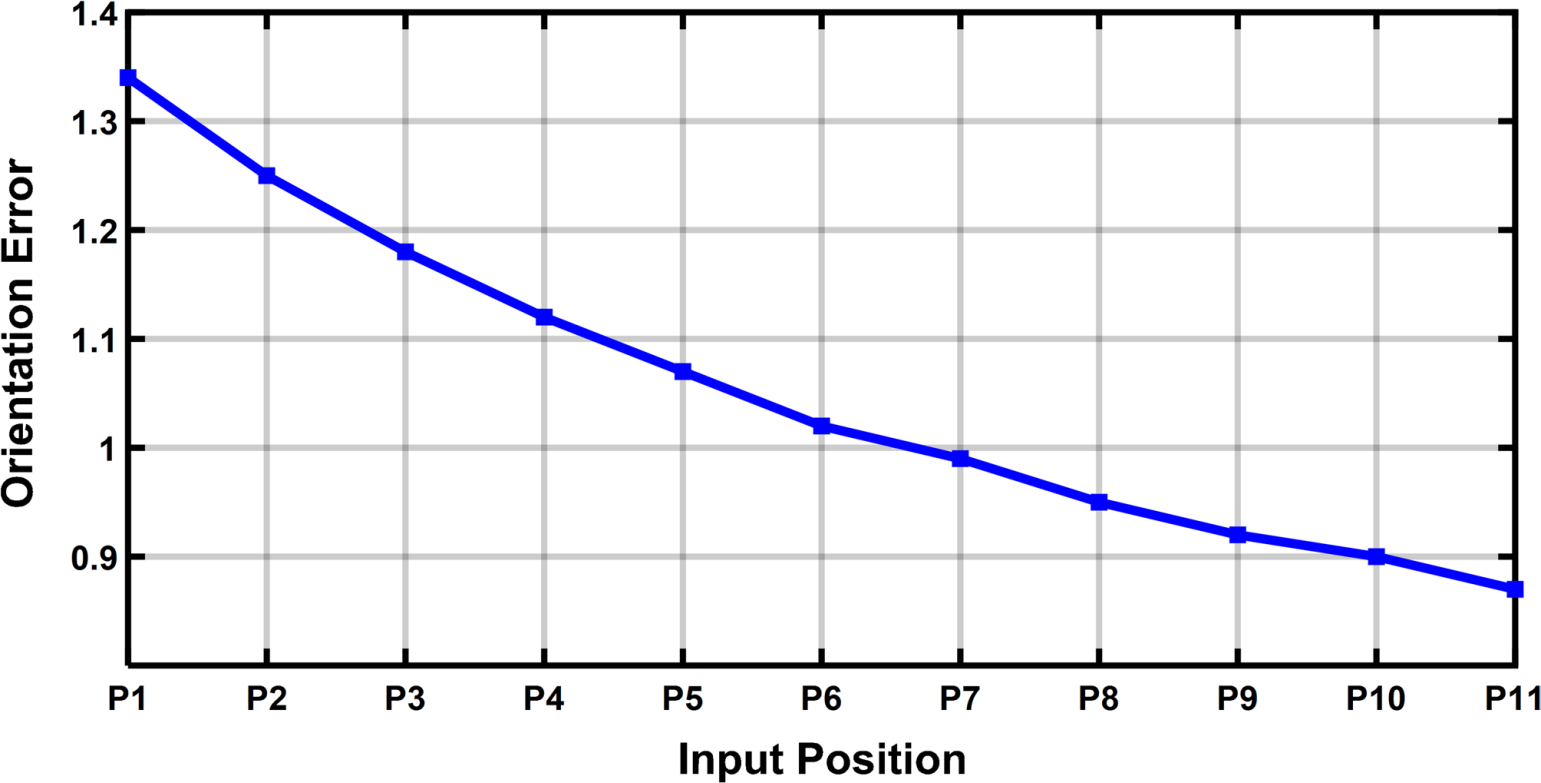
Input position vs Orientation Error (Radial Link 1).

**Fig. 15:**
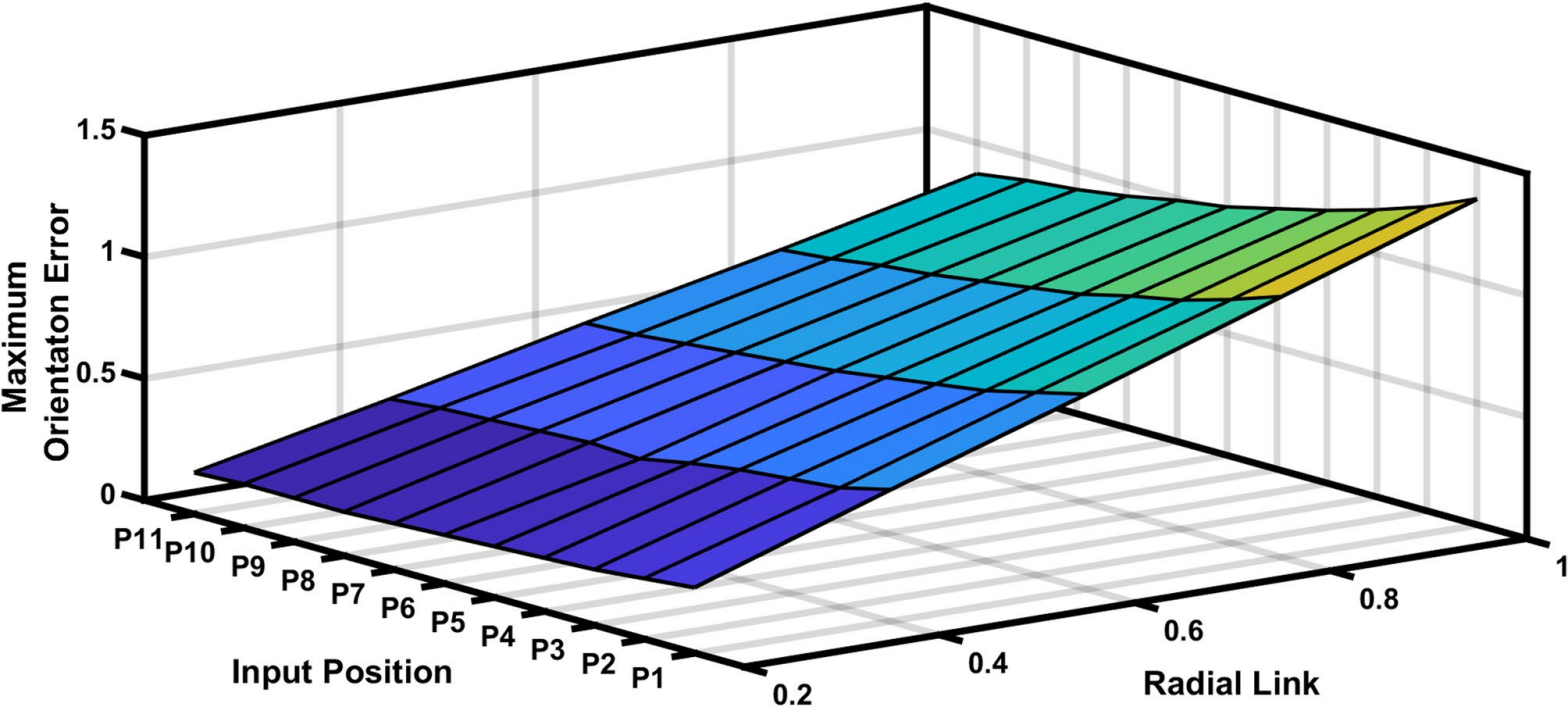
Input Position vs Radial Link vs Maximum Orientation Error.

Figure 13 represents the 3D surface plot of radial link vs coupler position vs estimated positional error, it signifies the increase in positional error with increase in level of clearances simultaneously indicating a constant maximum error for the given range radial link length. The Fig. 14 represents the maximum orientation error vs 11 input positions of the actuator for maximum radial link joint clearance of the 4R mechanism. The orientation error between the desired and deviated angle and the error is estimated and observed that the error is almost identical to 1. The Fig. 14 depicts the 2D plot of input position vs orientation error, it shows that the orientation error decreases as the input angle increased to be larger for input position. Figure 15 represents the surface plot of input position vs maximum orientation error for each given radial link length. As the radial link length increases, the optimal orientation error for each position increases simultaneously (Table 3).

**Table 3:** Maximum orientation error for radial link 1 mm.

Maximum orientation error 4R			
Position	$\theta_{3}$	$\theta_{{3\left( {deviated} \right)}}$	Orientation error
P1	35.57	34.23	1.34
P2	38.21	36.96	1.25
P3	40.82	39.64	1.18
P4	43.42	42.3	1.12
P5	46	44.93	1.07
P6	48.58	47.56	1.02
P7	51.16	50.17	0.99
P8	53.74	52.79	0.95
P9	56.34	55.42	0.92
P10	58.95	58.05	0.9
P11	61.57	60.7	0.87

### Error estimation on P-type mechanism

**Fig. 16:**
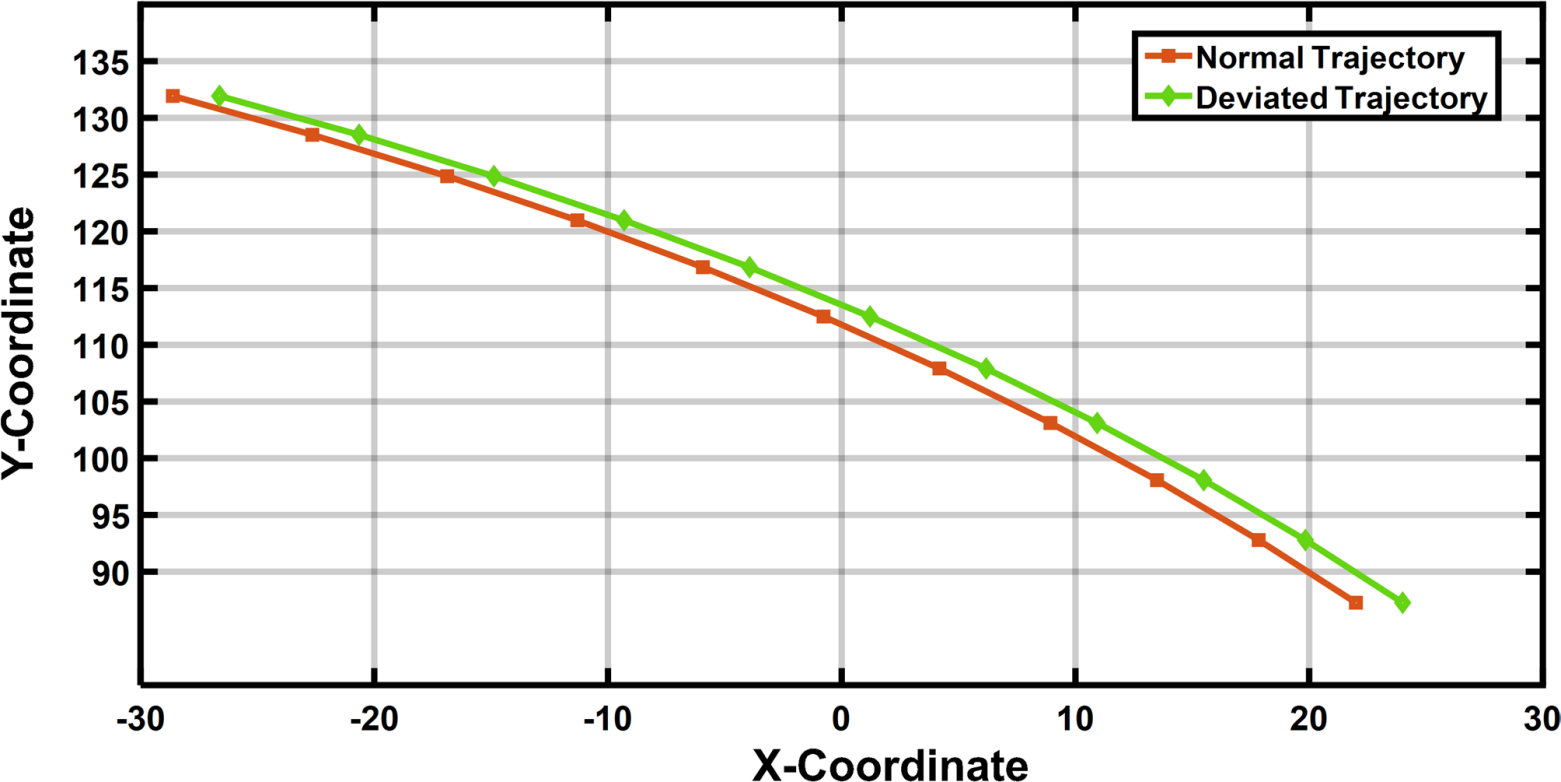
Coupler position in P3R mechanism.

Figure 16 depicts the normal and deviated trajectory of the coupler position in the P-type mechanism with joint clearance in the task space system. The maximum deviated coupler position for 11 linear actuator position is evaluated with the maximum clearance level [52]. There is a deviation in the trajectory generated by the P3R mechanism in Table 4.

**Table 4:** Deviated Trajectory for P3R Mechanism.

P3R mechanism						
S. No	Position	Input link position	Desired coupler position		Deviated coupler position	
			X Co-ordinate	Y Co-ordinate	X Co-ordinate	Y Co-ordinate
1	P1	90	22	87.27	24	87.27
2	P2	94.5	17.849	92.799	19.849	92.799
3	P3	99	13.495	98.076	15.495	98.076
4	P4	103.5	8.939	103.113	10.939	103.113
5	P5	108	4.18	107.919	6.18	107.919
6	P6	112.5	− 0.781	112.497	1.219	112.497
7	P7	117	− 5.945	116.849	− 3.945	116.849
8	P8	121.5	− 11.311	120.972	− 9.311	120.972
9	P9	126	− 16.88	124.864	− 14.88	124.864
10	P10	130.5	− 22.651	128.519	− 20.651	128.519
11	P11	135	− 28.625	131.93	− 26.625	131.93

**Fig. 17:**
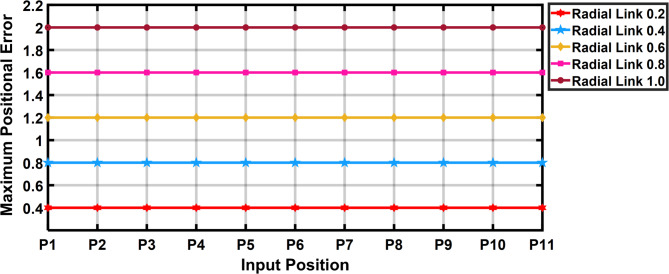
Input position vs Maximum positional error P3R mechanism.

**Fig. 18:**
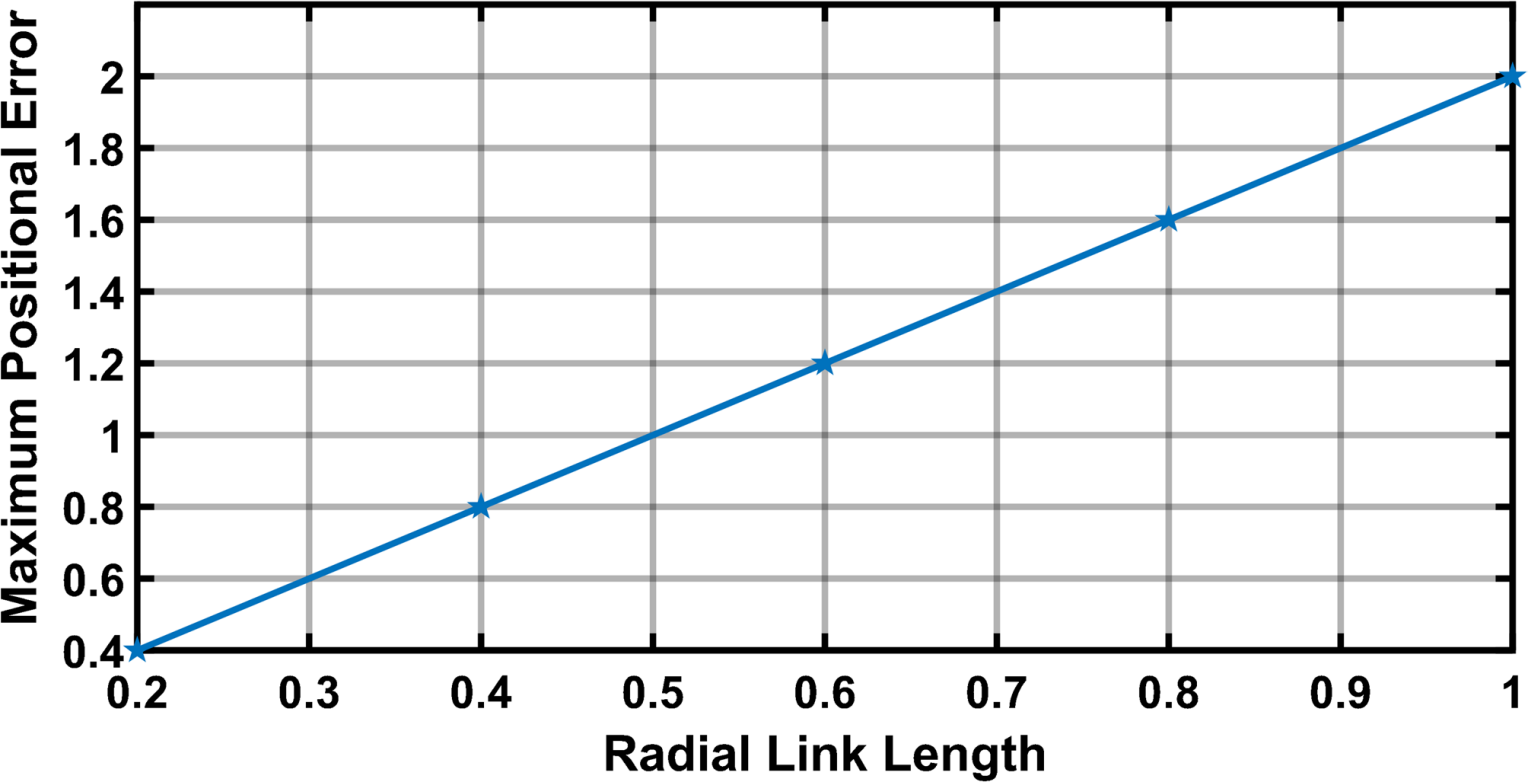
Radial link vs Maximum positional error P3R mechanism.

**Fig. 19:**
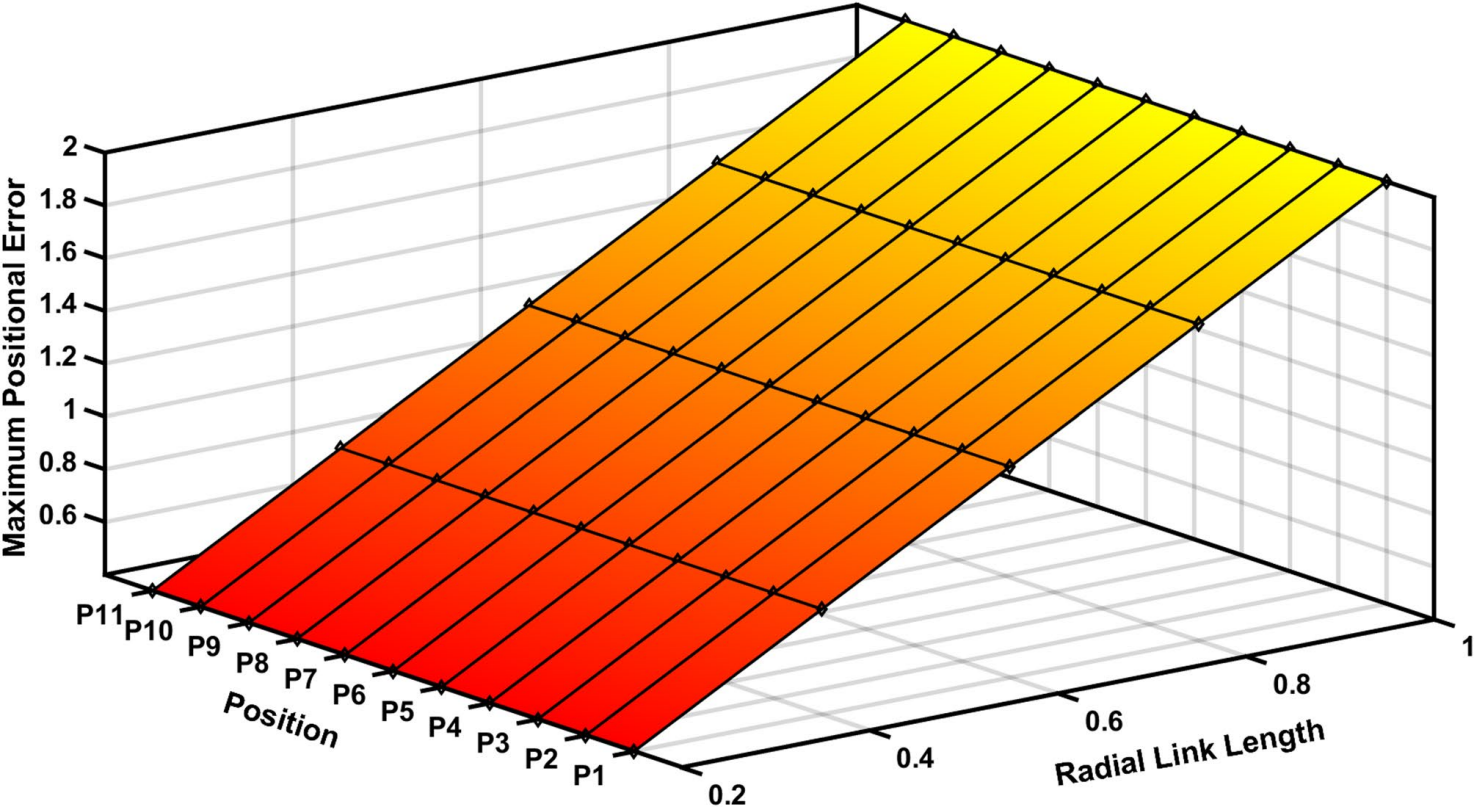
Input position vs radial link vs maximum positional error.

The Fig. 17 shows the input positions versus the maximum positional error generated for a radial link (joint eccentricity). The maximum positional inaccuracy for a given radial link length for a certain point is twice the radial link length, as indicated in Fig. 17. The maximum positional error is shown to be a constant for a considered radial link length in Fig. 17. However, the radial link was 1 mm in length, the highest positional error obtained is 2 mm, indicating the increase in estimated error with increase in joint clearances (Fig. 18). Figure 19 is a 3D surface plot of radial link vs. input position vs. maximum positional error, exhibiting an increase in maximum positional error with increasing radial link length while also indicating a constant maximum error for a given radial link length.

**Fig. 20:**
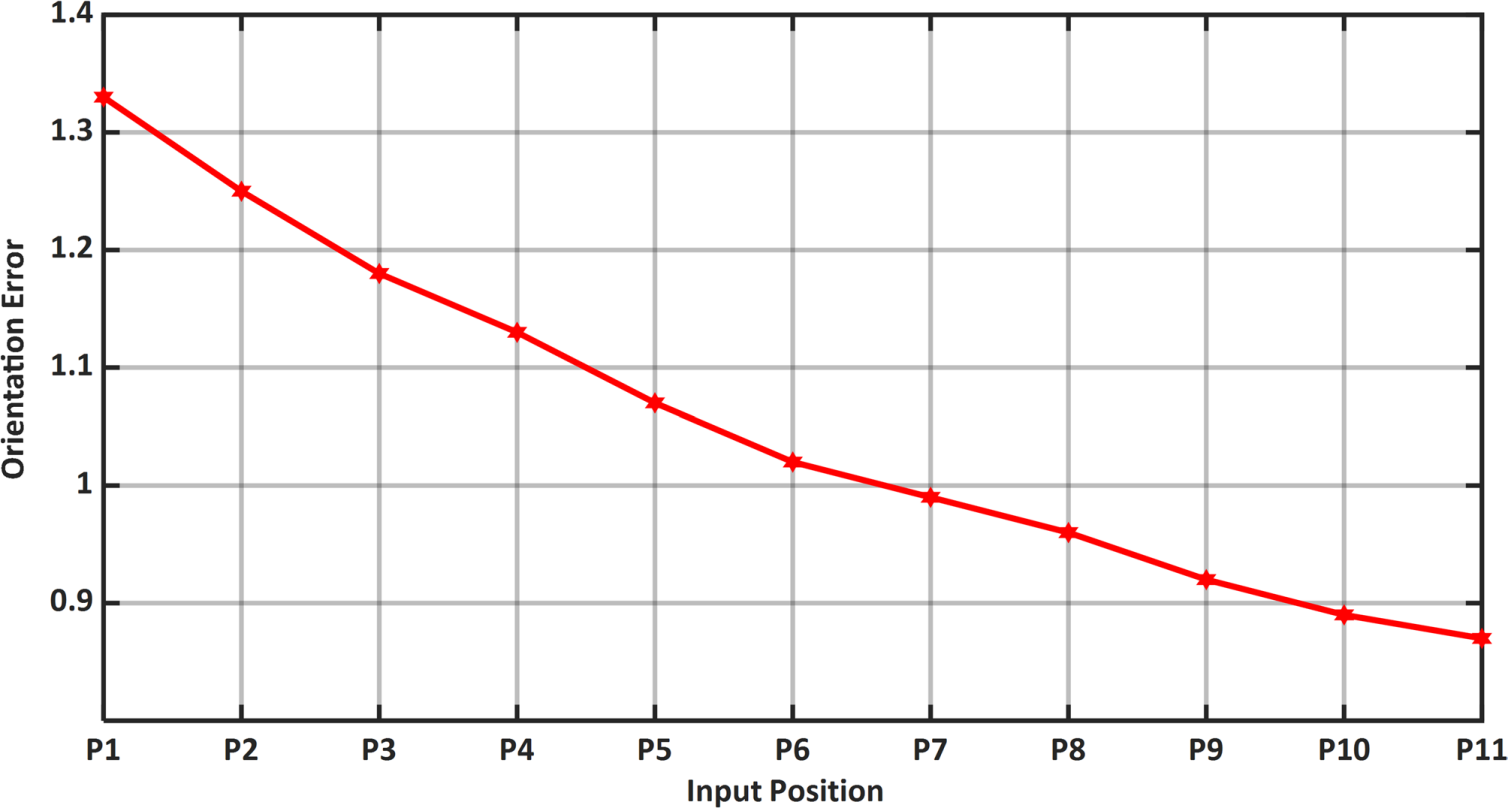
Input position vs Orientation error P3R mechanism.

**Fig. 21:**
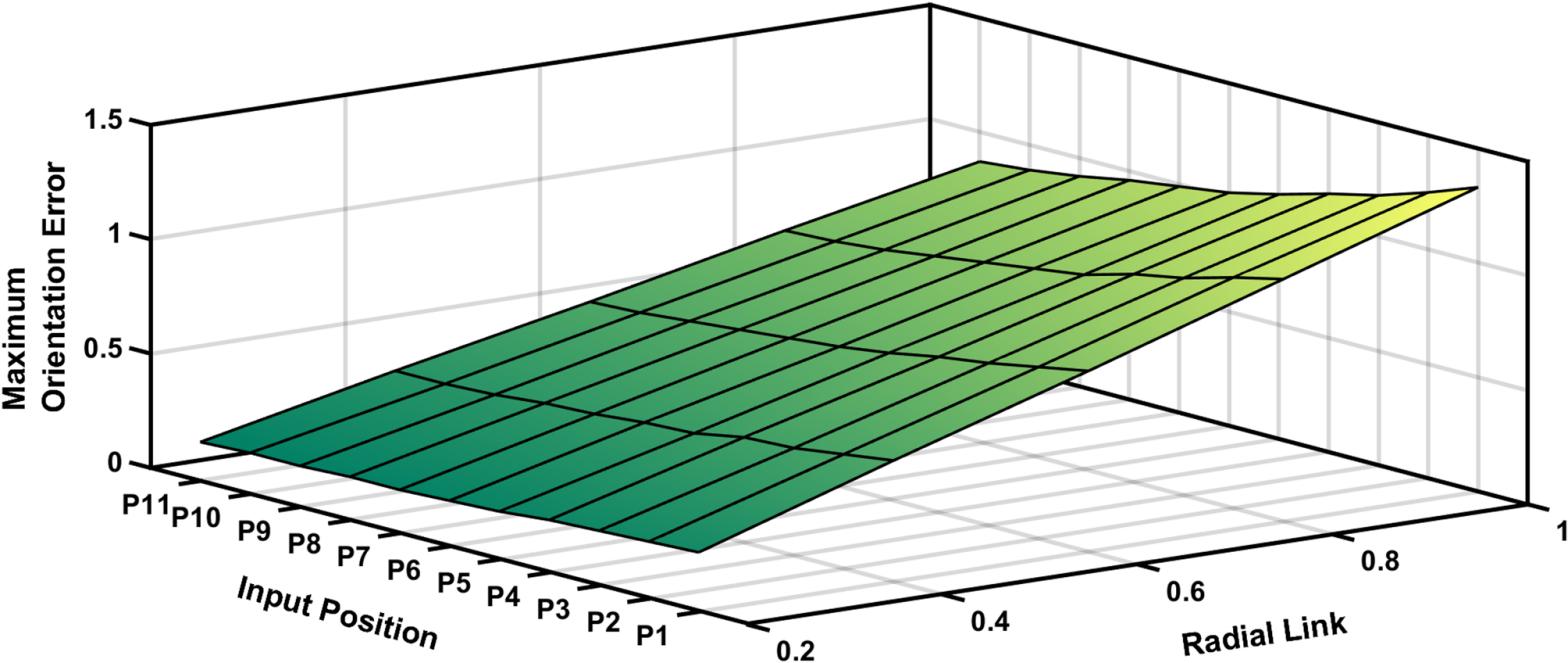
P3R Mechanism Orientation Error Plot.

The maximum orientation error recorded at the output link for each of the 11 input points is shown in Table 5. The desired and deviated coupler position is determined along with the error, and it is observed that the error is nearly equal to one, i.e. the maximum positional error is radial link length. The Fig. 20 depicts the 2D plot of input position versus orientation error, it shows that the orientation error decreases as the input angle decreased to be larger for input position. Figure 21 represents the surface plot of input position versus the maximum orientation error for each given radial link length. As the radial link length increases, the optimal orientation error for each position increases simultaneously.

**Table 5:** Maximum orientation error for radial link 1 mm.

Maximum orientation error P3R			
Position	$\theta_{3}$	$\theta_{{3\left( {new} \right)}}$	Orientation error
P1	35.57	34.24	1.33
P2	38.22	36.97	1.25
P3	40.83	39.65	1.18
P4	43.43	42.3	1.13
P5	46	44.93	1.07
P6	48.58	47.56	1.02
P7	51.16	50.17	0.99
P8	53.75	52.79	0.96
P9	56.34	55.42	0.92
P10	58.95	58.06	0.89
P11	61.58	60.71	0.87

### CAD model verification

**Fig. 22:**
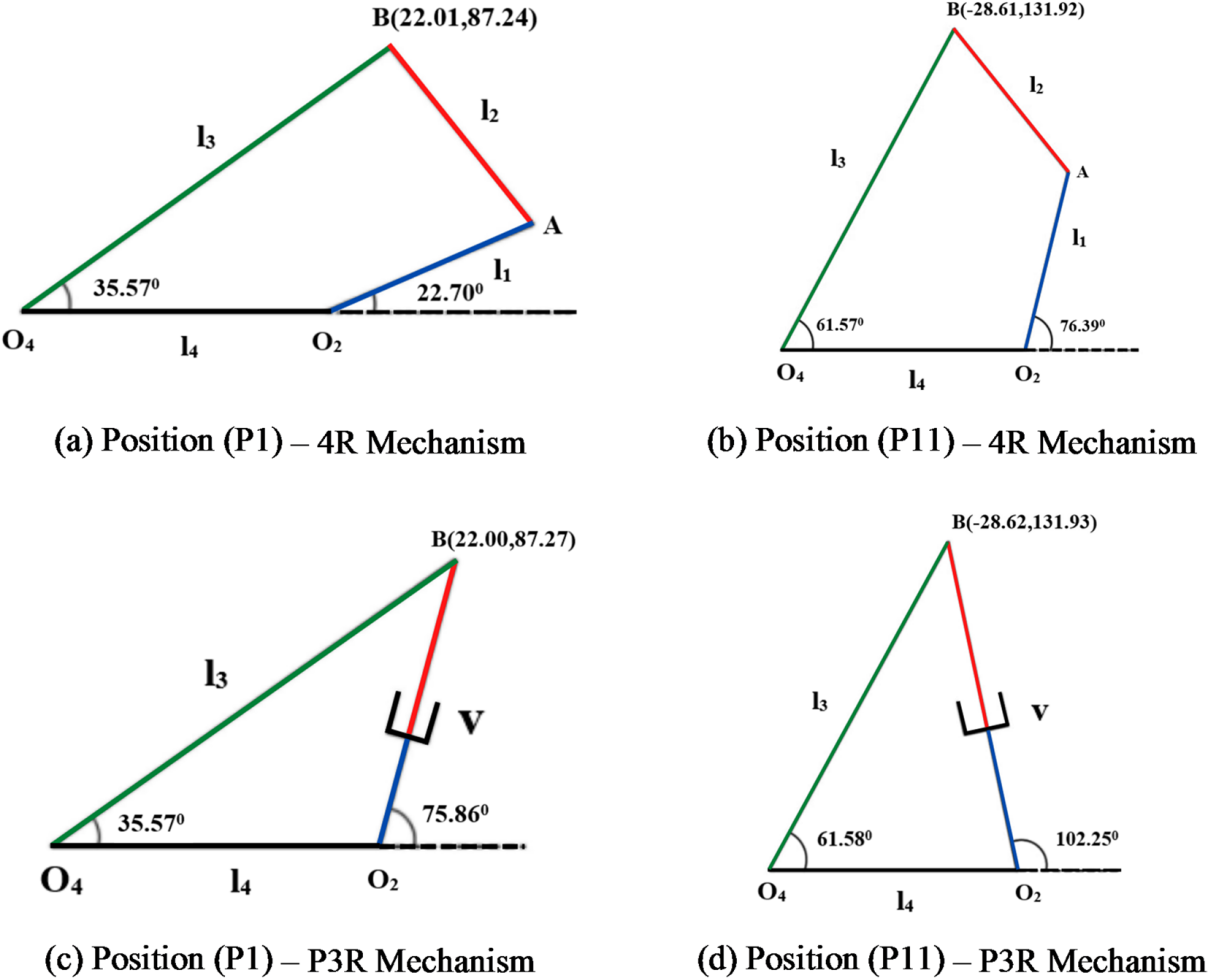
(a-d) Verification of position and orientation using CAD model approach.

The Fig. 22a, b and c, d shows the desired coupler position and orientation angle of the output link using the CAD model of the R-type and P-type mechanisms. The position and orientation of both mechanisms are examined through mathematical model results. The Table 6 represents the position of coupler and orientation angles for P1 and P11 using mathematical and CAD model approach.

**Table 6:** Comparison of the desired position and orientation between the mathematical model and CAD model.

Coupler position	4R mechanism						P3R mechanism					
	Mathematical Model			CAD Model			Mathematical Model			CAD Model		
	*B*_*x*_	*B*_*y*_	*θ*_3_º	*B*_*x*_	*B*_*y*_	*θ*_3_º	*B*_*x*_	*B*_*y*_	*θ*_3_º	*B*_*x*_	*B*_*y*_	*θ*_3_º
P1	22.015	87.248	35.57	22.01	87.24	35.57	22.00	87.27	35.57	22.00	87.27	35.57
P11	-28.615	131.925	61.57	-28.61	131.92	61.57	-28.625	131.93	61.58	-28.62	131.93	61.58

**Fig. 23:**
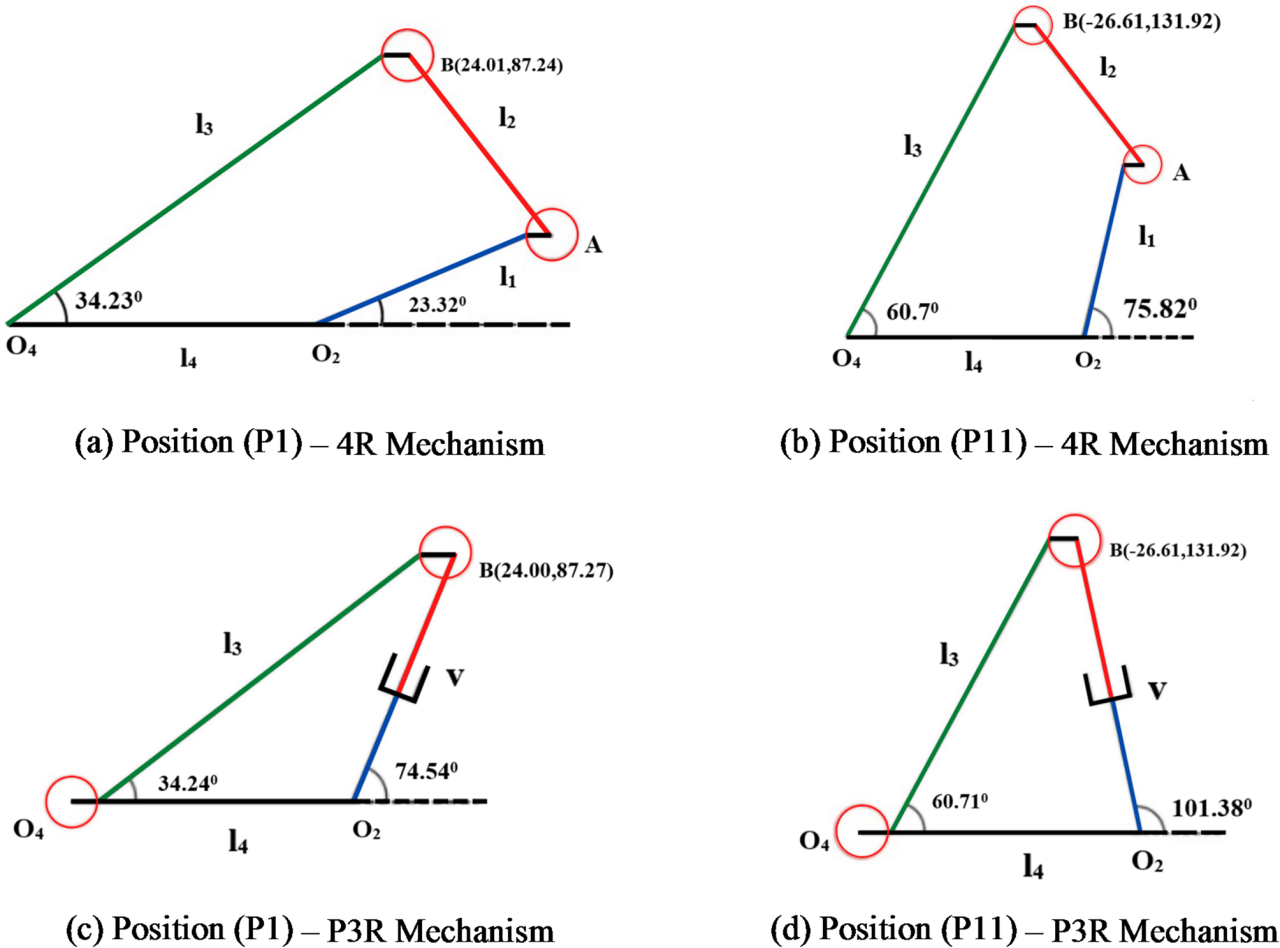
(a-d) Verification of position and orientation with joint clearance using CAD model approach.

The Fig. 23a, b and c, d shows the results of deviated position of coupler and orientation angle of the output link for the 4R and P3R mechanisms using the CAD model. The Table 7 represents the deviated coordinates of the coupler and orientation angles of both (4R and P3R) mechanisms through the formulated mathematical approach and CAD model are almost similar. A detailed CAD simulation was performed. Both desired and clearance-affected trajectories were replicated in the CAD environment. The resulting coupler positions and orientation angles from the CAD model were compared to the position and orientation obtained from the mathematical model to ensure consistency and accuracy.

**Table 7:** Comparison of the deviated coupler position and output angle between the mathematical model and CAD model.

Coupler position	4R mechanism with clearance						P3R mechanism with clearance					
	Mathematical model			CAD model			Mathematical model			CAD model		
	*B*_*x*_	*B*_*y*_	*θ*_3_º	*B*_*x*_	*B*_*y*_	*θ*_3_º	*B*_*x*_	*B*_*y*_	*θ*_3_º	*B*_*x*_	*B*_*y*_	*θ*_3_º
P1	24.015	87.248	34.23	24.01	87.24	34.23	24.00	87.27	34.24	24.00	87.27	34.24
P11	-26.615	131.925	60.7	-26.61	131.92	60.7	-26.625	131.93	60.71	-26.61	131.92	60.71

### Error compensation of R-type & P-type mechanisms

The angle of error compensation obtained through the inverse kinematics to compensate the maximum positional error in each input position (rotary actuator) due to joint clearance (1 mm radial link) for 4R and P3R mechanisms are determined by Tables 8 and 9. The compensation of the error is beneficial for the mechanism to regain its desired position.

**Table 8:** Error compensation of 4R mechanism (radial link 1).

Error compensation 4R mechanism			
Position	$\theta_{1}$	$\theta_{{1\left( {deviated} \right)}}$	Error compensation
P1	22.7	23.32	0.62
P2	28.16	28.62	0.46
P3	33.46	33.81	0.35
P4	38.67	38.88	0.21
P5	43.83	43.93	0.1
P6	48.98	48.99	0.01
P7	54.17	53.92	0.25
P8	59.43	59.23	0.2
P9	64.83	64.52	0.31
P10	70.45	70.01	0.44
P11	76.39	75.82	0.57

**Table 9:** Error compensation of P3R mechanism for radial link 1.

Error compensation P3R mechanism radial link 1			
Position	*v*	*v (deviated)*	Error compensation
P1	90	88.43	1.57
P2	94.5	93.03	1.47
P3	99	97.59	1.41
P4	103.5	102.1	1.4
P5	108	106.68	1.32
P6	112.5	111.24	1.26
P7	117	115.75	1.25
P8	121.5	120.28	1.22
P9	126	124.82	1.18
P10	130.5	129.33	1.17
P11	135	133.85	1.15

### Comparative study between 4R & P3R mechanism

**Fig. 24:**
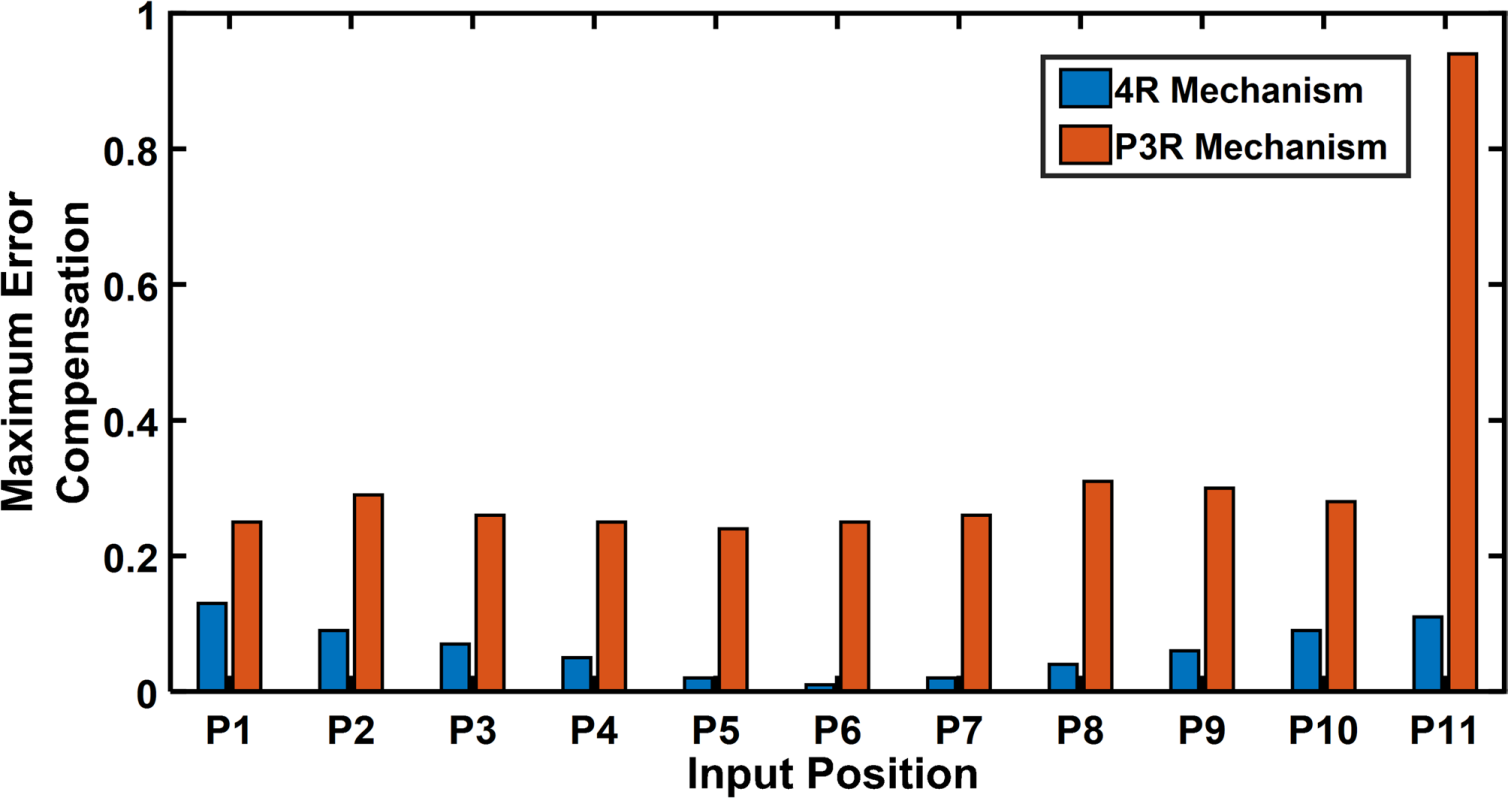
Input position vs Maximum error compensation (radial link 0.2).

**Fig. 25:**
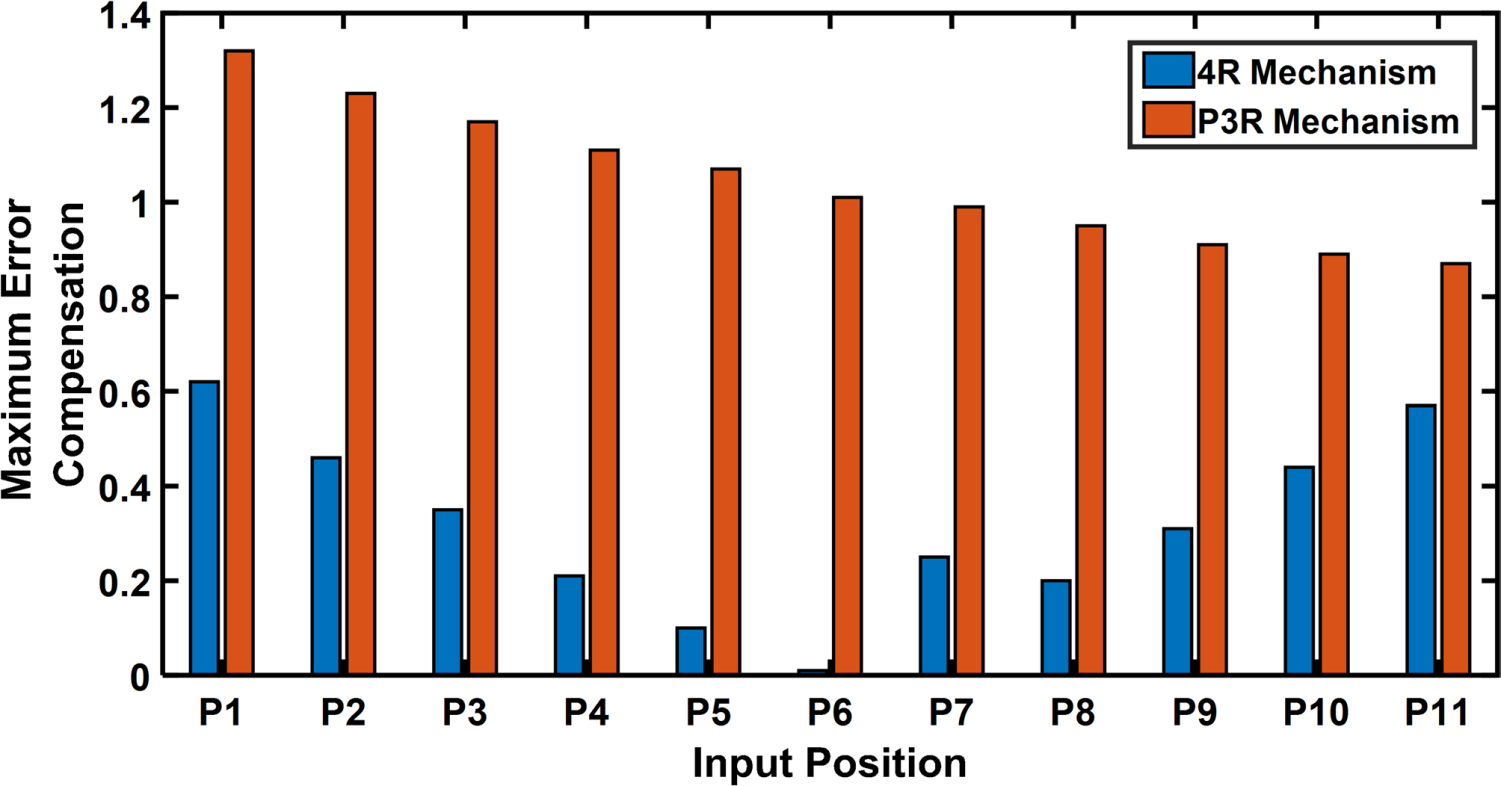
Input position vs Maximum error compensation (radial link 1).

**Fig. 26:**
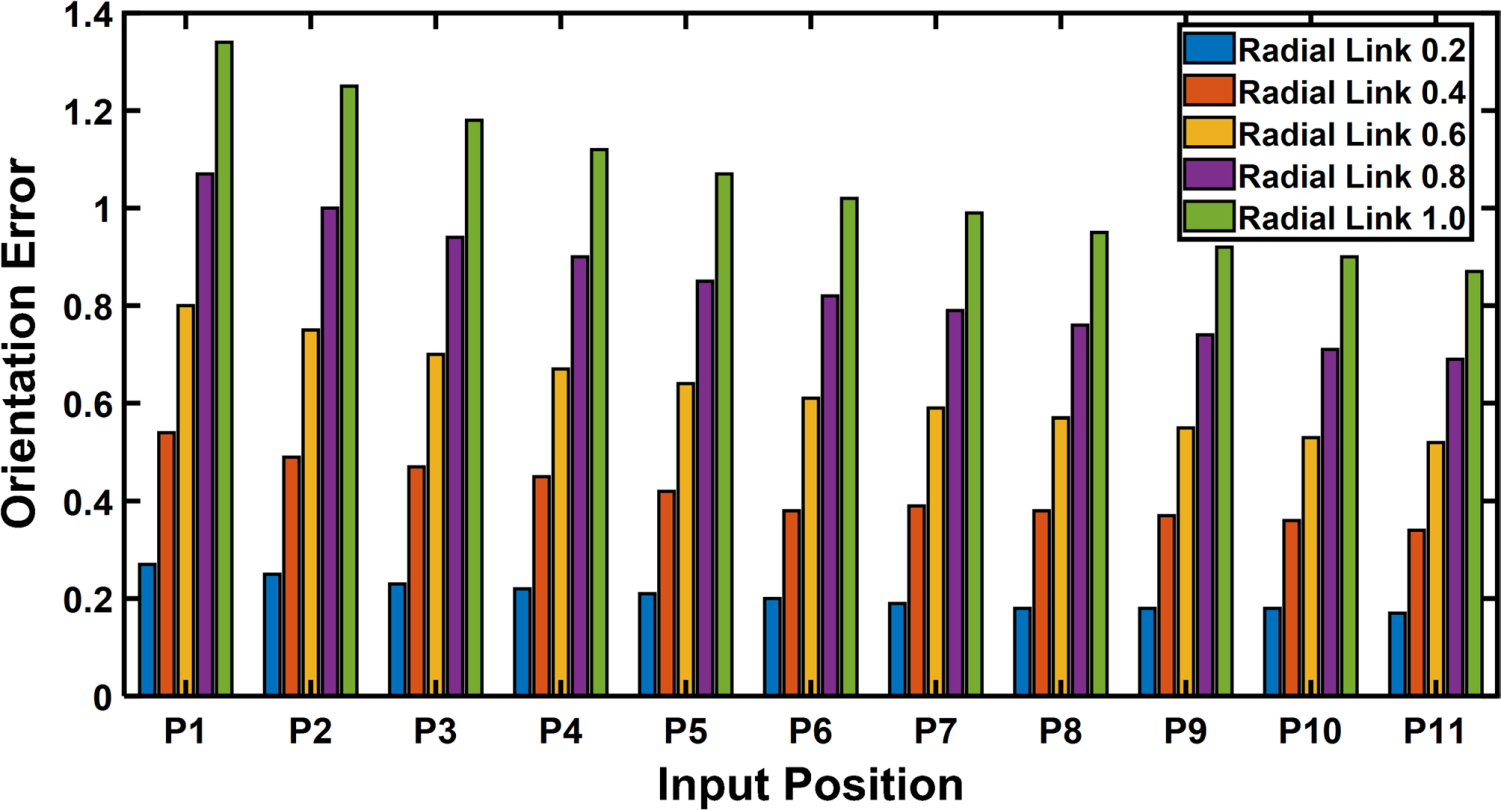
A 4R Mechanism – Input position vs Orientation Error.

**Fig. 27:**
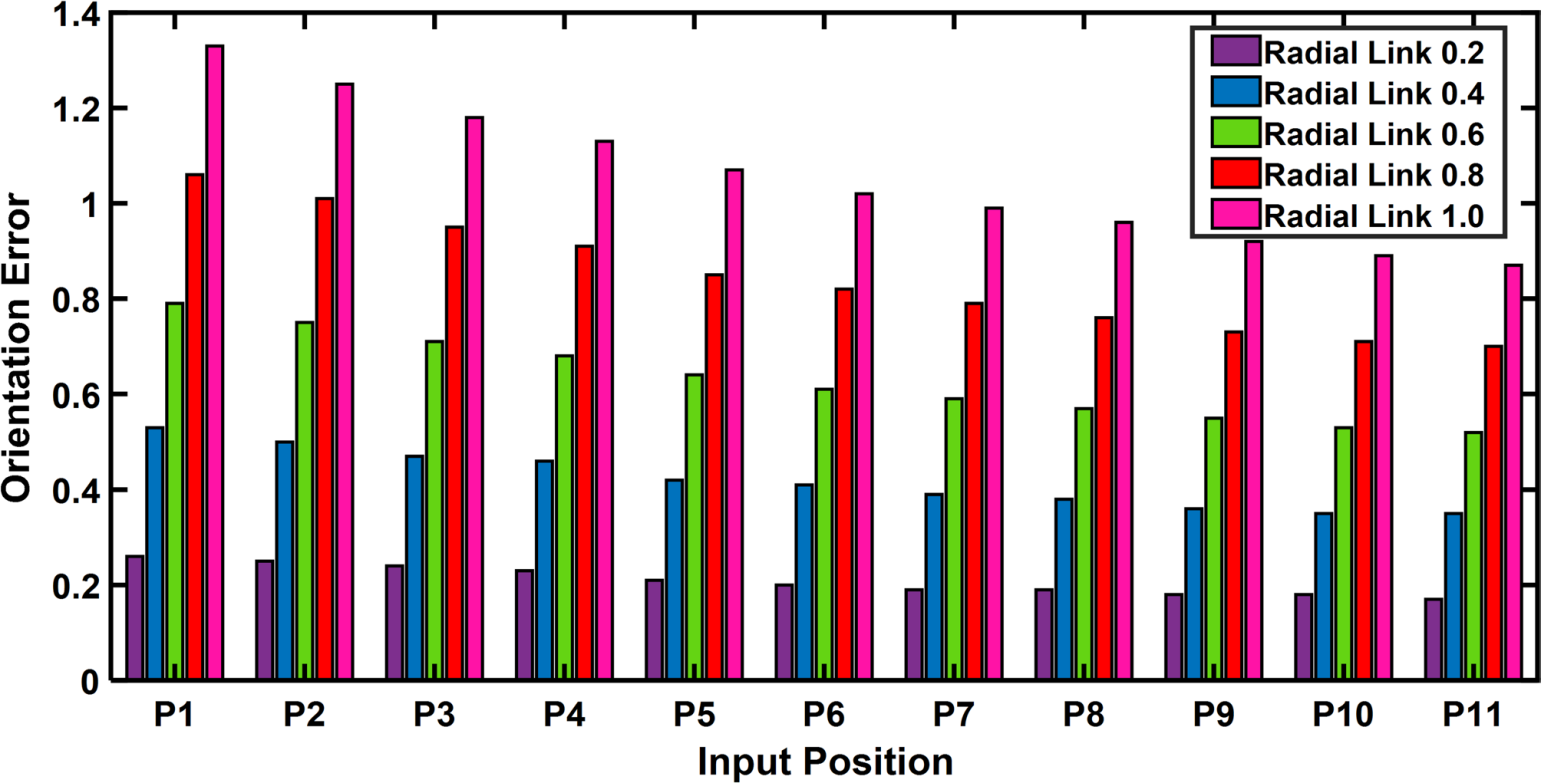
P3R Mechanism—Input position vs Orientation error.

The error compensation for a 4R and P3R mechanisms with a radial link length of 0.2 are shown in Tables 10 and 11. As shown by Fig. 24, a comparison shows that the error correction required for a 4R mechanism is significantly less than that of a P3R mechanism. Similarly, the error compensation for P3R and 4R mechanisms for each point at a radial link length of 1 (Fig. 25) shows that P3R requires more error compensation than 4R mechanism. The input position and maximum orientation error for a certain radial link length were compared (Figs. 26 and 27), demonstrating the 4R’s better positional accuracy.

**Table 10:** Error compensation of 4R mechanism for radial link 0.2

Error compensation 4R mechanism radial link 0.2			
Position	Theta 1	Theta 1 (D*eviated)*	Error compensation
P1	22.7	22.83	0.13
P2	28.16	28.25	0.09
P3	33.46	33.53	0.07
P4	38.67	38.72	0.05
P5	43.83	43.85	0.02
P6	48.98	48.99	0.01
P7	54.17	54.15	0.02
P8	59.43	59.39	0.04
P9	64.83	64.77	0.06
P10	70.45	70.36	0.09
P11	76.39	76.28	0.11

**Table 11:** Error compensation of P3R mechanism for radial link 0.2

Error compensation P3R mechanism radial link 0.2			
Position	*v*	*v' (Deviated)*	Error Compensation
P1	90	89.75	0.25
P2	94.5	94.21	0.29
P3	99	98.74	0.26
P4	103.5	103.25	0.25
P5	108	107.76	0.24
P6	112.5	112.25	0.25
P7	117	116.74	0.26
P8	121.5	121.19	0.31
P9	126	125.7	0.3
P10	130.5	130.22	0.28
P11	135	134.06	0.94

This section compares the error compensation required for 4R and P3R mechanisms under joint clearance at radial link lengths of 0.2 and 1.0. As shown in Tables 10 and 11, the 4R mechanism exhibits significantly lower error compensation (0.01°–0.13°) compared to the P3R mechanism (0.24–0.94 units) for the same trajectory points (P1–P11).

Figure 24 illustrates this difference clearly for a link length of 0.2, with the P3R mechanism consistently requiring higher compensation. This trend is further confirmed at a longer link length (Fig. 25), where clearance effects are amplified, but the 4R still maintains better positional stability.

Orientation error plots (Figs. 26 and 27) also show that the 4R mechanism maintains lower and more consistent orientation error across the input range, while the P3R exhibits greater fluctuations.

## Conclusions

This study’s main contribution is to examine the maximum positional inaccuracy of the 4R and P3R mechanisms when joint clearances are present. Simultaneously finding the most suitable mode of actuation i.e. prismatic or rotation for selection of robot drives. The mechanisms were studied under identical or equivalent operating conditions and having studied them, subsequent broad conclusions are derived based on the study. Firstly, throughout the whole working range (P1 to P11), the mechanical error due to joint clearances for both mechanisms varies continuously. The error is often assumed to be uniform throughout the trajectory. It was concluded that positional inaccuracy in the trajectory and all clearing links is equivalent to the same amount. Lastly, comparative analysis of positional error under the effect of joint clearance in 4R and P3R mechanisms shows that, under similar operating conditions, the 4R design displays more robustness than the P3R configuration. Thus, it is demonstrated that revolving joint actuation of robotic manipulators becomes preferable to prismatic actuation from the perspective of positional error sensitivity.

It will be beneficial to utilize the developed approach and reported performance to investigate parallel mechanisms with R-type and P-type mechanisms. Future research will focus on expanding the ideas and methodology employed in this work to examine further issues with mechanism performance.

## Data Availability

The data used and/or analyzed during the current study are available from the corresponding author upon reasonable request.

## List of symbols

*l*_1_, *l*_2_, *l*_3_ And *l*_4_: Link Lengths: input link, coupler link, output link and fixed link respectively
*θ*_1_, *θ*_3_: Input angular displacement and output angular displacement
*δ*_1_, *δ*_2_ and *δ*_3_: Radial length of the mechanism (i.e. Joint Clearance)
λ_1_, λ_2_ And λ_3_: Angle of the radial length
*v*: Displacement of the linear actuator
